# The endometrial cancer A230V-ALK5 (TGFBR1) mutant attenuates TGF-β signaling and exhibits reduced *in vitro* sensitivity to ALK5 inhibitors

**DOI:** 10.1371/journal.pone.0312806

**Published:** 2024-11-22

**Authors:** Eun-Jeong Yu, Daphne W. Bell

**Affiliations:** Reproductive Cancer Genetics Section, Cancer Genetics and Comparative Genomics Branch, National Human Genome Research Institute, National Institutes of Health, Bethesda, Maryland, United States of America; Ondokuz Mayis University Faculty of Medicine: Ondokuz Mayis Universitesi Tip Fakultesi, TÜRKIYE

## Abstract

The ALK5 (TGFBR1) receptor serine/threonine kinase transduces TGF-β (Transforming Growth Factor beta) signaling to activate SMAD2/3-dependent and -independent pathways. Here, we aimed to determine the functional consequences of *ALK5* mutations in human endometrial cancer (EC). Somatic mutation data were retrieved from publicly available databases. Using seven *in silico* algorithms, 78.5% (11 of 14) of ALK5 kinase domain mutations in EC, including A230V-ALK5, were predicted to impact protein function. For *in vitro* studies, we focused on A230V-ALK5 because it was the only mutated residue located within the ATP-binding pocket, which is an important region for both ATP-binding and binding of ATP-competitive inhibitors. Constructs expressing wildtype-, constitutively-active-, kinase-dead-, or mutant A230V-ALK5, were transfected into NIH/3T3 cells. Following TGF-β1 stimulation, transient exogenous expression of A230V-ALK5 resulted in attenuated SMAD2/3 signal transduction and reduced AKT activation. We further showed that the A230V-ALK5 mutant had reduced stability resulting from increased ubiquitin-dependent protein degradation. Our structural modeling predicted that SB-431542, a small molecule ATP-competitive inhibitor of ALK5, binds to the A230V-ALK5 mutant with reduced affinity compared to wildtype-ALK5. We therefore examined the inhibitory effect of SB-431542 and galunisertib on wildtype- and mutant-ALK5 activity using a Smad-binding element (SBE) luciferase reporter assay combined with TGF-β1 stimulation, in NIH/3T3 cells and HEC-265 EC cells. SBE luciferase activity in A230V-ALK5 transfected cells was inhibited less by SB-431542 and galunisertib than in wildtype-ALK5 transfected cells indicating that A230V-ALK5 is less sensitive to inhibition by these agents than wildtype-ALK5, potentially due to changes in SB-431542/A230V-ALK5 binding affinity. Our findings are novel and show that A230V-ALK5 is a partial loss-of-function mutant that attenuates TGF-β1 signal transduction and has reduced sensitivity to ALK5 small molecule inhibitors.

## Introduction

ALK5 (TGFBR1) and TGFBR2 are receptor serine-threonine kinases that mediate TGF-β-induced signal transduction via SMAD2-dependent (canonical) and SMAD2-independent (non-canonical) pathways [1]. Signal transduction is initiated upon binding of the TGF-β ligand to TGFBR2, within a heteromeric complex with ALK5. Activated TGFBR2 then phosphorylates the GS domain of ALK5, which relieves inhibition of ALK5 by the inhibitory FKBP12 protein. Once activated, ALK5 phosphorylates SMAD2 and SMAD3, facilitating formation of a SMAD2/3/4 heterotrimer. The SMAD2/3/4 complex then translocates from the cytoplasm into the nucleus where it regulates transcription of TGF-β target genes. Non-canonical SMAD-independent signaling pathways that are activated in response to TGF-β stimulation include the PI3K/AKT, MEK/ERK, and MKK/JNK/MAP pathways.

ALK5 plays important roles in the normal physiology of the murine uterus [2–4]. Several recently described genetically engineered mouse models have shown that ALK5 is a tumor suppressor in the endometrium. Conditional inactivation of *Alk5* in progesterone-expressing tissues, including the murine endometrium, results in the development of estrogen-dependent metastatic endometrial cancer (EC) but only in the animals mated continuously to wildtype males for at least 6-months [5]. Moreover, concurrent conditional inactivation of *Alk5* and *Pten* in progesterone-expressing tissues results in metastatic EC [6], in contrast to *Pgr-cre*-mediated *Pten* deletion which results in the development of primary, but not metastatic, EC.

EC is currently the 5^th^ leading cause of cancer death among women in the US [7], but is expected to become the 4^th^ leading cause of such deaths by 2040 [8]. *ALK5* is somatically mutated in a subset of human ECs [9]. Because *Alk5*-deficiency promotes the development of metastatic EC in a genetically engineered mouse model and ALK5 is druggable [10], it is important to understand the functional consequences of *ALK5* mutations that occur in human EC and their effect on *in vitro* sensitivity to ALK5 inhibitors.

In this study we functionally characterized the ALK5-A230V mutation, because among the residues mutated in the ALK5 kinase domain in EC, A230 is the only mutation located within the ATP-binding pocket, which is an important subdomain for both ATP-binding and binding of ATP-competitive inhibitors. Moreover, a different mutation (A230T) at position 230 is a pathogenic germline mutation that causes Loeys-Dietz syndrome (LDS), thus highlighting the importance of the A230 residue for proper functioning of ALK5. In the era of personalized medicine, it is important to understand the functional consequences of individual mutations within druggable kinases, particularly those that affect sites of kinase-inhibitor interactions. For all of these reasons combined, we prioritized the A230V mutation for analysis. In the era of personalized medicine, it is important to understand the functional consequences of individual mutations within druggable kinases, particularly those affect sites of kinase-inhibitor interactions. Herein, we show that the A230V-ALK5 kinase domain mutation, which occurs in EC, encodes a partial loss-of-function protein that is less stable than wildtype ALK5. The reduced stability of the A230V-ALK5 mutant is attributable, at least in part, to increased ubiquitination and more rapid proteasomal degradation than wildtype ALK5. We further demonstrate that compared to wildtype-ALK5, the A230V-ALK5 mutant is less sensitive to *in vitro* inhibition by SB-431542 and galunisertib, two ATP-competitive small molecule inhibitors of ALK5. These findings are novel and provide mechanistic insights into the functional consequences of mutant ALK5 in human EC.

## Methods

### Mutation identification

To identify somatic mutations of *ALK5* in endometrial carcinomas, we used the cBioPortal for Cancer Genomics (https://cbioportal.org) [11, 12] to query publicly available mutation datasets using the search term “*TGFBR1*”. The queried datasets were previously generated by The Cancer Genome Atlas (TCGA) or Memorial Sloan-Kettering Cancer Center (MSKCC) and corresponded to the following cohorts: TCGA-Uterine Corpus Endometrial Carcinoma (PanCancer) [13], MSKCC-Advanced-Stage Endometrial Cancer [14], MSKCC-Endometrial Cancer MSI (MSK, 2022) [15], and TCGA-Uterine Carcinosarcoma (PanCancer) [16]. Publicly available clinicopathologic information for *ALK5*-mutated cases was also retrieved from the cBioPortal for Cancer Genomics.

### Predicting the functional impact of ALK5 missense mutations

*ALK5* missense mutations were subjected to in silico analyses to obtain the predicted impact on protein function. The following prediction tools were used: SIFT (Sorting Intolerant From Tolerant) [17], MutationAssessor (Release 3) [18], PolyPhen-2 (Polymorphism Phenotyping v2) [19], PMut2017 [20], SNAP2 (Screening for Non-Acceptable Polymorphisms, version 2) [21], PANTHER (Protein ANalysis THrough Evolutionary Relationships) classification system (version 16.0) [22], REVEL (Rare Exome Variant Ensemble Learner) [23].

### Molecular docking and binding energy calculations

Molecular docking and binding energy calculations were carried out using AutoDock Vina (‘Vina’) software (Scripps Research, La Jolla, CA) [24–26], utilizing the computational resources of the NIH High Performance Computing Biowulf cluster (https://hpc.nih.gov). The X-ray structure of ALK5 in complex with SB-431542 (PDB: 3TZM) was retrieved from the RCSB Protein Data Bank (RCSB PDB, rcsb.org), and PubChem (https://pubchem.ncbi.nlm.nih.gov/) was used to download the 3D structure of ligands (SB-431542: CID 4521392; ATP: CID 5957). The PDB file was loaded into PyMOL Molecular Graphics System Version 2.0 (Schrödinger, LLC) and the sequence change (alanine>valine) on the alanine 230 (A230) residue of ALK5 was incorporated using the mutagenesis tool provided in the software.

To run Vina receptor and ligand docking predictions, both the receptor and ligand molecules were prepared for docking by removing water molecules and adding hydrogen atoms to the structure using AutoDockTools (ADT) from MGLTools (Scripps Research, La Jolla, CA) and then converted into pdbqt format. For protein-ligand docking, a configuration file was created using MGLTools which contains all the necessary information required for docking. The AutoDock scoring function was utilized to calculate binding energy and affinity between receptor and ligand molecules. Docking run input files were written in a text file for execution of the Vina run, and a completed log and output file in pdbqt form was generated once the docking was completed. All modified molecules were docked into the active site on TGFβRI (PDB ID: 3TZM) to obtain the docking score, binding affinity, and estimated Ki (inhibitory constant) value.

### Cell lines and cell culture

The research conducted in this study was excluded from IRB Review per the Common Rule 45 CFR 46 and NIH policy for the use of specimens/data. The murine NIH/3T3 cell line was purchased from the American Type Culture Collection (ATCC) (Manassas, VA) and maintained in DMEM(ATCC)+ 10% bovine calf serum (ATCC). The HEC-265 endometrioid EC cell line [27] was purchased from the Japanese Collection of Research Bioresources (JCRB) cell bank (Osaka, Japan) (JCRB No: JCRB1142) and maintained in EMEM (ATCC) + 15% bovine calf serum.

Cell line authentication was performed at the beginning of the study using short tandem repeat profiling as a fee-for-service by ATCC (for NIH/3T3) or Laragen Inc., (Culver City, CA) (for HEC-265). Cell lines were verified as being mycoplasma-free with the MycoAlert mycoplasma detection kit (Lonza, Rockland, ME) using the MycoAlert assay control set (Lonza) according to the manufacturer’s instructions. All cell cultures were maintained at 37°C in a humidified atmosphere with 5% CO_2_. Passage numbers did not exceed 20 for NIH/3T3 or HEC-265 cells; passage-1 was defined as the first passage in this laboratory after cell line procurement.

### Mammalian expression constructs

The following N-terminal-3xFLAG-tagged ALK5 expression constructs were generated by GenScript (Piscataway, NJ), as a fee-for-service, using DNA synthesis followed by cloning into the pCMV-3Tag-1A vector: ALK5_wild-type_ pCMV-3Tag-1A (abbreviated WT-ALK5), ALK5_A230V_ pCMV-3Tag-1A (abbreviated A230V-ALK5), ALK5_T204D_ pCMV-3Tag-1A (constitutively active control, abbreviated T204D-ALK5), ALK5_K232R_ pCMV-3Tag-1A (kinase-dead control, abbreviated K232R-ALK5). The sequence of the wild-type *ALK5* open-reading frame (ORF) corresponds to NM_004612.4. The ALK5 p.A230V, p.T204D, and p.K232R ORFs include, respectively, the following mutations: c.689C>T (p.A230V), c.610AC>GA (p.T204D), c.695A>G (p.K232R). The pCMV-3Tag-1A vector was obtained from Agilent Technologies (Santa Clara, CA). The pGL4.48[SBE] luciferase reporter constructs and the pGL4.74[TK] Vector internal control construct were purchased from Promega (Madison, WI). The N-terminal HA-tagged pCMV6-AN-HA_Ubiquitin expression construct was a gift from Roger Woodgate (Addgene plasmid # 131258; http://n2t.net/addgene:131258; RRID: Addgene_131258).

### Plasmid DNA isolation

For ALK5 expression constructs, transfection grade DNA (≥95% Supercoiled, ≤0.005 EU/μg Endotoxin) was prepared by GenScript. Sanger sequencing was used to verify the integrity of the entire insert of all ALK5 constructs; primer sequences and sequencing conditions are available on request. Empty vector DNA was isolated from bacterial cultures using the QIAGEN Plasmid Plus Midi Kit (Qiagen, Valencia, CA) according to the manufacturer’s instructions.

### Transient transfection, ligand stimulation, and drug treatment

For transient transfection, cells were plated in a 12-well plate (5 x 10^4^ cells/well [NIH/3T3] or 2 x 10^5^ cells/well [HEC-265]) and incubated at 37°C in 5% CO_2_ for 24 h prior to transfection of 0.5 μg of ALK5 constructs for NIH/3T3 cells, or 1 μg of constructs for HEC-265 cells using LipofectAMINE 3000 (Invitrogen, Waltham, MA) at 1:2 DNA/lipofectamine ratio in 100 μl of Opti-MEM™ I Reduced-Serum Medium (Gibco, Waltham, MA).

Recombinant human TGF-beta(β)1 (Human Cell-expressed) Protein (R&D systems, Minneapolis, MN) was reconstituted at 100 μg/mL in sterile 4 mM HCl containing 0.1% bovine serum albumin. Serum-free cell culture media was used for further dilutions to give final concentrations of 0.1 ng/mL—1 ng/mL. The small molecule inhibitors SB-431542 and galunisertib were purchased from Tocris Bioscience (Bristol, UK) and dissolved in dimethylsulfoxide (DMSO) at a concentration of 10 mM. Further dilutions of an inhibitor were made in serum-free cell culture media before applying to cell cultures for a final concentration of 0.25 μM-8 μM. Cells were serum-starved in serum-free medium for 30 min before treatment with either TGF-β1 ligand, inhibitor, or vehicle.

### Luciferase reporter assays

Luciferase reporter assays were performed using the Dual-Glo® Luciferase assay system (Promega) according to the manufacturer’s protocol. For transient transfection, cells were plated in a 48-well plate (1x 10^4^ cells/well [NIH/3T3] or 4 x 10^4^ cells/well [HEC-265]) and incubated at 37°C in 5% CO_2_ for 24 h before transfection. Each experimental condition had five replicated wells. Cells were triply transfected with an ALK5 expression construct (50 ng), pGL4.48[SBE] Firefly luciferase reporter construct (100 ng), and pGL4.74[TK] Renilla vector (10 ng) as an internal control construct to control for variations in transfection efficiency at a final volume of 20 μl. After 24 h transfection, cells were stimulated with TGF-β1 for 3 h and Dual-Glo® Luciferase solution was added to measure firefly luminescence. After 20 min incubation, cells and luciferase solution were transferred to a 96-well white flat bottom assay plate and firefly luminescence was measured using the FLUOstar Omega microplate reader (BMG Labtech, Cary, NC) in luminescence detection mode and the 3600-gain setting. To measure Renilla luminescence, the Dual-Glo® Stop & Glo® reagent was added to the same plate and incubated for 20 min before measurement.

Normalization was performed by calculating the ratio of Firefly: Renilla luminescence counts from the plate reading for each well. To test the effect of a small molecule inhibitor, transfected cells were treated with an inhibitor or vehicle (DMSO) at the previously described concentrations for one hour before 3 h ligand stimulation. The entire experiment was repeated four times and SBE luciferase activity measurement for each sample was used to calculate the mean ± SD, and the statistical analyses were performed using GraphPad Prism 10 (Graph-Pad Software, Boston, MA).

### Western blotting and preparation of whole-cell extracts

For western blots, with the exception of the co-immunoprecipitation (co-IP) experiments, cells were plated in a 12-well plate and transfected as described above. After 24 h incubation and treatment with the corresponding ligand, inhibitor, or vehicle, cells were lysed on ice using RIPA buffer (Thermo Fisher Scientific, Waltham, MA) containing protease inhibitor cocktail (Cell Signaling Technology (CST), Danvers, MA). After boiling for 5 min at 95°C in the presence of 4X Laemmli sample buffer, aliquots containing equal amounts of total protein were resolved by electrophoresis on a 10% (w/v) or 4–15% gradient SDS-polyacrylamide gel (Bio-Rad, Hercules, CA) and subjected to western blotting using the following antibodies: DYKDDDDK Tag Mouse mAb (CST, #8146), Phospho-SMAD2 (Ser465/Ser467) rabbit mAb (CST, #18338), Smad2 Rabbit mAb (CST, #5339), recombinant rabbit anti-Smad3 (phospho S423 + S425) (abcam, Waltham, MA, #ab52903), Smad3 rabbit mAb (CST, #9523), Phospho-Akt (Ser473) rabbit mAb (CST, #4060), Phospho-Akt (Thr308) Rabbit mAb (CST, #13038), Pan-Akt rabbit mAb (CST, #4691), Phospho-mTOR (Ser2448) rabbit mAb (CST, #5536), mTOR rabbit mAb (CST, #2983), rabbit polyclonal Anti-TGF beta Receptor I antibody (Abcam, #ab31013), and Anti-β-Actin mAb (Sigma-Aldrich, #A2228).

To investigate more than one target protein on the same blot, we used Restore™ PLUS Western Blot Stripping Buffer (Thermo Fisher Scientific) for removing bound primary and secondary antibodies from membranes and reprobed with a primary antibody for the next target. For detection of western blot signals, we used Clarity or Clarity Max ECL chemiluminescent substrates (Bio-Rad) and exposed the membrane to blue autoradiography film.

### Co-immunoprecipitation under denaturing conditions

For co-IP experiments, lysates from transfected cells were prepared using 1% NP-40 buffer solution consisting of 20 mM Tris [pH 8], 137 mM NaCl, 10% [v/v] glycerol, 1% [v/v] NP-40, 2 mM EDTA (ethylenediaminetetraacetic acid), 1X protease inhibitor cocktail. DYKDDDDK-Tag rabbit mAb Sepharose Bead Conjugate (CST, #70569) was used to capture FLAG-ALK5 protein overnight at 4°C on a rotator, and the bead-immune complex was washed using the same lysis buffer. To elute, 2X Laemmli sample buffer was added to the complex directly and boiled at 95°C for 5 min. After a quick centrifugation, the entire sample was loaded on a 10% (w/v) SDS-polyacrylamide gel (Bio-Rad). The following antibodies were used for western blotting: DYKDDDDK Tag Mouse mAb (CST, #8146), HA-Tag Mouse mAb (CST, #2367), Ubiquitin Mouse mAb (CST, #3936), K48-linkage Specific Polyubiquitin Rabbit mAb (CST, #8081), K63-linkage Specific Polyubiquitin Rabbit mAb (CST, #5621).

### Protein stability analysis

To conduct cycloheximide chase experiments, cells were seeded in a 12-well plate, incubated for 24 h and then *ALK5* expression constructs or empty vector were transiently transfected as described above “transient transfection”. After 24 h incubation, cycloheximide (Calbiochem, St. Louis, MO), dissolved in DMSO, was added to give a final concentration of 50 μg/mL and, at the indicated time points, cells were harvested for immunoblotting. To inhibit proteasome activity, after 24 h transfection, cells were pretreated with MG-132 (Calbiochem) in DMSO at a final concentration of 25 μM for 3h followed by 50 μg/mL of cycloheximide treatment for 15 min before cell lysate collection.

## Results

### Incidence and spectrum of *ALK5* somatic mutations in EC

We used the cBioPortal for Cancer Genomics [11, 12] to query the *ALK5* mutation status of tumors in the TCGA-UCEC and TCGA-UCS datasets [12, 15], after excluding the TCGA-UCEC ultramutated subgroup, and in the MSKCC clinical cohorts [13, 14]. Overall, 27 somatic mutations in *ALK5* were identified among the 907 endometrial tumors queried (S1 Fig); 40.7% (11 of 27) of mutations, including the recurrent S241L-, R255C-, and R487G-ALK5 mutations, localized within the protein kinase domain, and 14.8% (4 of 27) were in the Activin types I and II receptor domain. The majority (81.5%, 22 of 27) of mutations were missense mutations; the remainder were splice-site (7.4%, 2 of 27), nonsense (7.4%, 2 of 27), or in-frame deletion (3.7%, 1 of 27) mutations.

In the TCGA datasets, *ALK5* was mutated in 2.7% (13 of 351) of non-ultramutated endometrioid ECs, 0.9% (1 of 107) of serous ECs, and 0% (0 of 56) uterine carcinosarcomas. In non-ultramutated endometrioid ECs, *ALK5* mutations were found in all tumor grades: 14.3% (2 of 14) of grade 1; 21.4% (3 of 14) of grade 2; and 64.3% (9 of 14) of grade 3 tumors. When the TCGA-UCEC data were stratified by molecular subgroup, *ALK5* mutations were present in 8.7% (13 of 148) of MSI/hypermutated, 0% (0 of 147) of MSS/copy number low, and 0.6% (1 of 163) of copy number high ECs. Mutations were present in 7.2% (13 of 181) of recurrent or advanced ECs in the MSKCC clinical cohort.

### A subset of ALK5 missense mutations in EC is predicted, *in silico*, to impact protein function

*ALK5* mutations in EC could either be driver mutations that play a functional role in promoting the neoplastic process or inconsequential passenger mutations. We utilized a combination of seven *in silico* tools to determine the predicted functional impact of the 19 unique *ALK5* missense mutations in EC (Table 1). Thirteen missense mutations are predicted to impact protein function by at least 5 of 7 algorithms. In total, 78.5% (11 of 14) of unique missense mutations in the kinase domain (residues 207–490) are predicted to impact protein function compared to 40% (2 of 5) of mutations in other regions (Table 1).

**Table 1:** *In silico* predictions of functional impact for *ALK5* missense mutations found in endometrial cancer.

Nucleotide change		Amino acid change		Prediction tool						
				SIFT	Mutation Assessor	PMut2017	SNAP2	PolyPhen-2	Panther	REVEL
**Activin types I and II receptor domain**								Damaging		
	c.149C>T		p.T50I*	Damaging	Medium	Disease	Effect	Probably	Probably	0.927
	c.199C>T		p.H67Y	Tolerated	Low	Neutral	Neutral	Benign	Possibly	0.441
	c.284T>C		p.V95A	Tolerated	Low	Neutral	Effect	Benign	Possibly	0.123
	c.319A>G		p.N107D*	Damaging	Medium	Disease	Effect	Probably	Probably	0.817
	c.452G>A		p.R151H	Tolerated	Low	Neutral	Effect	Benign	Probably	0.119
**Kinase domain**										
	c.673C>T		p.R225W*	High	High	Disease	Effect	Probably	Probably	0.68
	c.689C>T		p.A230V*	Damaging	Medium	Disease	Effect	Probably	Probably	0.966
	c.695A>C		p.K232T*	Damaging	High	Disease	Effect	Probably	Probably	0.981
	c.722C>T		p.S241L*	Damaging	Medium	Disease	Effect	Probably	Probably	0.814
	c.763C>T		p.R255C*	Damaging	Medium	Disease	Effect	Probably	Probably	0.825
	c.790G>A		p.A264T*	Damaging	Low	Disease	Effect	Probably	Probably	0.9
	c.935G>A		p.G312D*	Damaging	High	Disease	Effect	Probably	Probably	0.961
	c.1064C>T		p.A355V*	Damaging	Medium	Disease	Effect	Probably	Probably	0.836
	c.1133A>G		p.Y378C*	Damaging	High	Disease	Effect	Probably	Probably	0.986
	c.1159T>A		p.S387T	Tolerated	Neutral	Neutral	Neutral	Benign	Probably	0.358
	c.1238G>A		p.R413Q*	Damaging	Low	Disease	Effect	Probably	Probably	0.786
	c.1328A>C		p.K443T	Damaging	Low	Disease	Effect	Possibly	Probably	0.334
	c.1347G>T		p.K449N	Tolerated	Neutral	Disease	Neutral	Benign	Probably	0.343
	c.1460G>A		p.R487Q*	Damaging	Low	Disease	Effect	Probably	Probably	0.9

*A missense mutation predicted to impact protein function by at least 5 of 7 algorithms

The mutations shown here are in part based upon data generated by the TCGA Research Network: https://www.cancer.gov/tcga" and retrieved using the cBIOPortal for Cancer Genomics (https://cbioportal.org). *ALK5* coding (c.) and ALK5 amino acid (p.) designations corresponding to CCDS6738.

### Molecular modeling predicts the A230V-ALK5 mutant has weakened interactions with ATP and the small molecule inhibitor SB-431542

**Fig 1:**
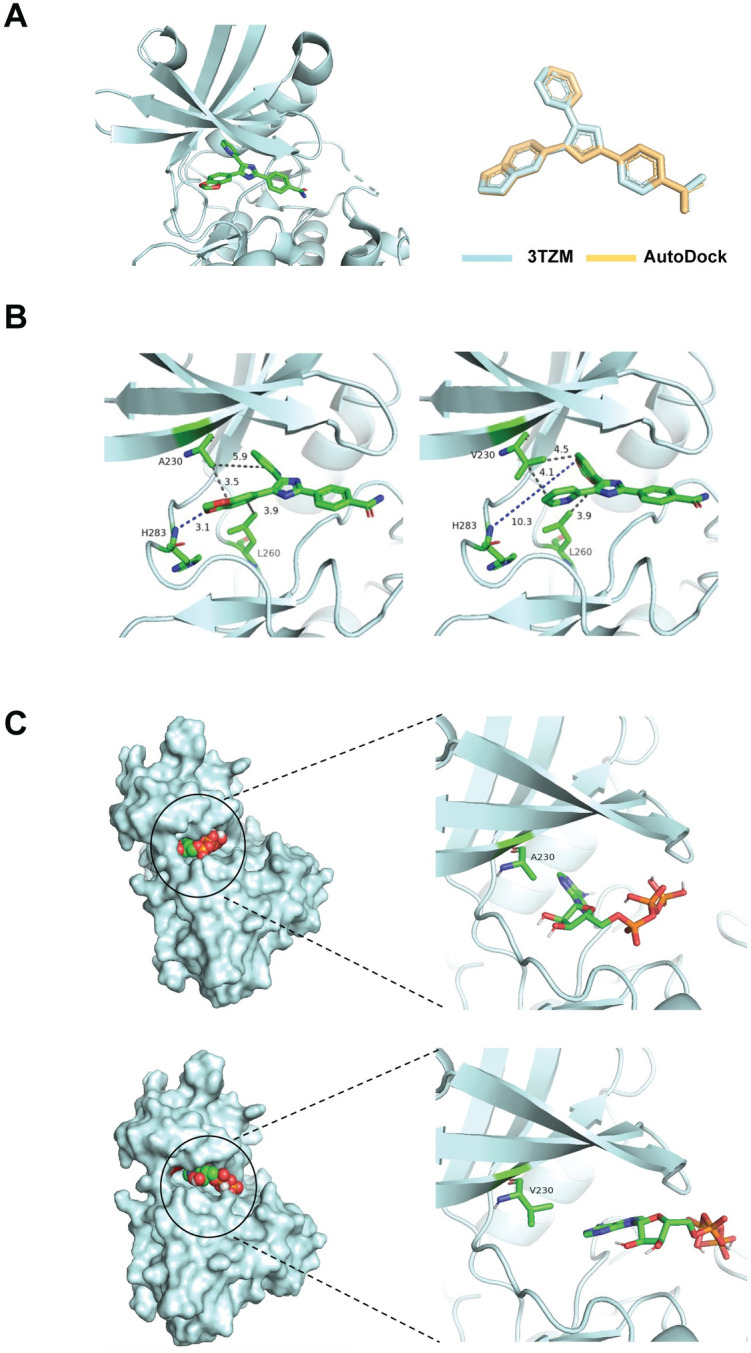
Molecular modeling of WT-ALK5 or A230V-ALK5 docked with SB-431542 or with ATP. The top-ranked pose for each docked state, as determined by AutoDock Vina docking simulations, is shown. (A) Crystal structure of TβRI-SB43154 complex (PDB:3TZM) (left) and validation pose (right) generated by re-docking of SB-431542 to WT-ALK5 (turquoise), and corresponding alignment pose of SB-431542 within the complex (gold). (B) Docking poses of WT-ALK5 (left) and the A230V-ALK5 mutant (right) with the SB-431542 inhibitor. (C) Docking poses of WT-ALK5 (top) and A230V-ALK5 (bottom) with ATP (PDB:1B0U). Dashed lines indicate angstrom distances between ALK5 residues and SB-431542 or ATP.

We chose to functionally evaluate the A230V-ALK5 kinase domain mutation because it affects an alanine residue that is located within the ATP-binding pocket and forms a hydrophobic interaction with the ATP-competitive small molecule SB-431542 [28]. To further predict how the A230V-ALK5 mutation might affect protein function, AutoDock Vina molecular docking software was used to analyze the conformation and orientation of small molecules, specifically ATP or SB-431542, into the wildtype (Alanine 230 (A230)) and mutant (Valine 230 (V230)) ALK5 receptors. To validate the accuracy of our docking protocol, we first removed SB-431542 from the PDB TGFBR1(ALK5)/SB-431542 complex (PDB: 3TZM) (Fig 1A, left), then re-docked it and calculated the root mean square deviation (RMSD) between the two poses after superimposing them (Fig 1A, right). Our results showed that the RMSD value between the docking pose and eutectic pose of SB-431542 in the active pocket of 3TZM was 1.067 Å, which confirmed that our AutoDock Vina protocol can be applied to obtain a reliable docking pose of ATP or SB-431542 and the ALK5 proteins. When SB-431542 was docked into the wildtype (A230) and mutant (V230) ALK5 receptor molecules, the distances between residue 230 and the benzodioxol- and pyridinyl-rings of the inhibitor were similar for both A230-ALK5 and V230-ALK5. However, compared with the wildtype-ALK5/SB-431542 complex (Fig 1B, left), the mutant-ALK5/SB-431542 complex had a rotation of the pyridinyl and benzodioxol ring portion of SB-431542 (Fig 1B, right), resulting in a 7.2 Å increase in the distance between the inhibitor and residue-283 of ALK5, thus making the V230-ALK5 mutant receptor inaccessible to the benzodioxol oxygen and resulting in loss of hydrogen bond formation between V230-ALK5 and SB-431542 (Fig 1B). When an ATP molecule was docked into the receptor molecules, the mutant V230 residue (Fig 1C, bottom) was located farther from ATP than the wildtype A230 residue was (Fig 1C, top). This change in ATP positioning could be due to the larger valine side chain filling the binding pocket of the mutant receptor and making the binding site inaccessible to ATP. Based on this structural prediction, we hypothesized that the A230V mutation will inhibit ALK5 kinase activity by disrupting its accessibility to ATP and will destabilize binding to SB-431542 as a result of weakened hydrogen bonds (S1 Table).

### *A230V-ALK5* is a partial loss-of-function mutation that attenuates TGF-β-induced activation of a SMAD-responsive reporter construct

**Fig 2:**
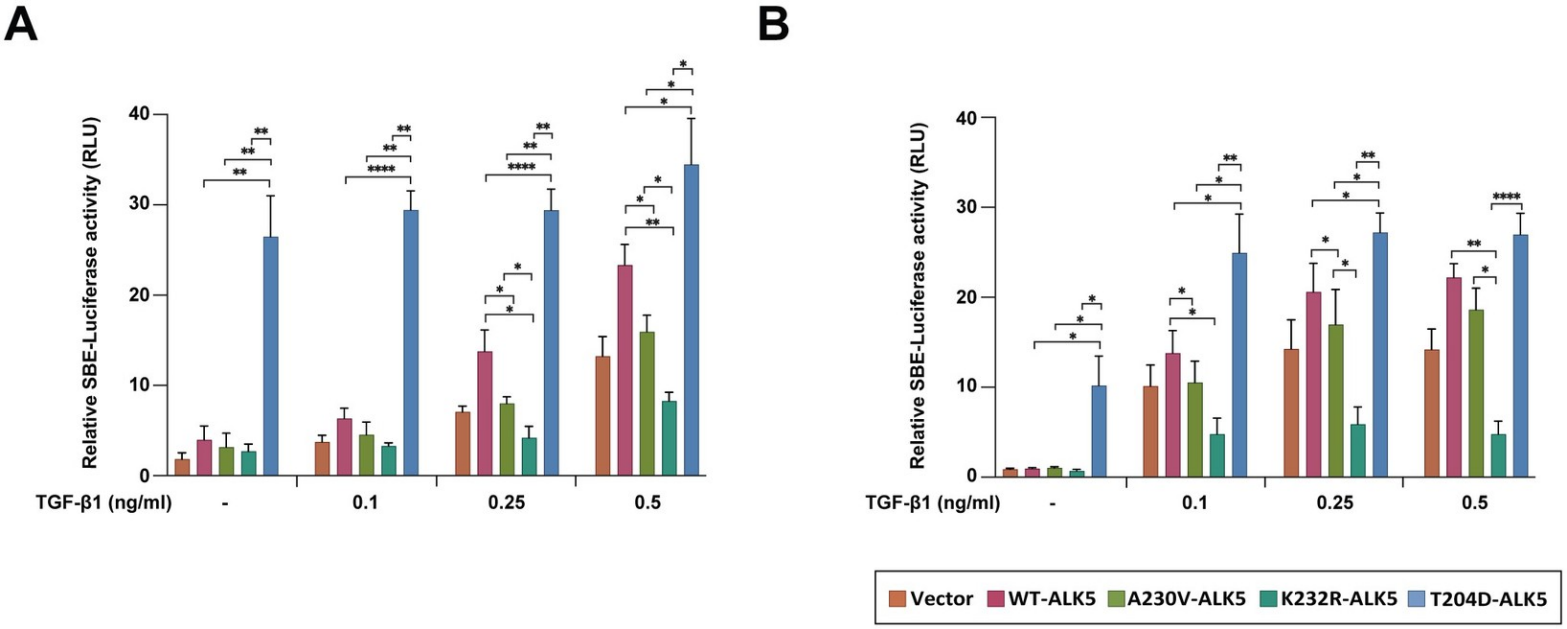
The A230V-ALK5 mutant behaves as a partial loss-of-function mutant in luciferase reporter assays following TGF-β1 stimulation. Results of TGF-β1-responsive SMAD binding element (SBE) firefly luciferase reporter assays in (A) NIH/3T3 cells, and (B) HEC-265 EC cells. Cells were triply transfected with pGL4.48[SBE] Firefly luciferase reporter construct, pGL4.74[TK] Renilla vector and either a wildtype (WT) or mutant FLAG-tagged ALK5 expression construct or control vector (CV). Expression constructs containing the T204D-ALK5 (constitutively-active mutant) or K232R-ALK5 (kinase-dead) mutants were included as positive and negative controls. After 24 h transfection, cells were serum-starved for 30 min before TGF-β1 was added at the indicated concentrations for an additional 3 h and luciferase activity was measured after the addition of assay reagents based on manufacturer’s protocol (Dual-Glo luciferase assay system). Relative Luciferase Units (RLU) were calculated by normalizing SBE luciferase activity to Renilla luciferase activity. The mean ± SD values, based on results of four independent experiments, were calculated using Prism software (GraphPad, Boston, MA) and plotted. P-values were calculated using Two-Way ANOVA; *P<0.05.

We next sought to test our hypothesis that the A230V mutation inhibits ALK5 kinase activity. We performed *in vitro* luciferase assays to compare the ability of FLAG-tagged wild-type and A230V-mutant ALK5 expression constructs to activate a SMAD-responsive luciferase reporter construct following transient transfection into NIH/3T3 mouse embryonic fibroblast cells (Fig 2A), which have low endogenous ALK5 protein levels.

The T204D-ALK5 (constitutively-active) and K232R-ALK5 (kinase-dead) constructs were included as controls [29]. The pGL4.74 Renilla vector (hRluc/TK promoter) was included as an internal control to normalize the luciferase reporter readout. Our results showed that in the absence of ligand stimulation, the relative luciferase activity of T204D-ALK5 transfectants was almost 6.65-fold higher than that of WT-ALK5 transfectants (Fig 2A), consistent with the constitutively active nature of the T204D-ALK5 mutant control [25]. In contrast, there were no significant differences in the relative luciferase activities of A230V-ALK5 or K232R-ALK5 transfectants compared to WT-ALK5 transfectants in unstimulated conditions. However, following stimulation with increasing concentrations of TGF-β1 ligand, K232R-ALK5 transfectants exhibited lower relative luciferase activity than either WT-ALK5 or vector-only transfectants, suggesting that the K232R mutation inhibits the ligand-dependent activity of endogenous ALK5 in NIH/3T3 cells, consistent with the kinase-dead/dominant-negative nature of this mutation [26]. Compared to WT-ALK5, the A230V-ALK5 mutant showed significantly lower relative luciferase activity in response to ligand (0.25ng/mL or 0.5 ng/mL) stimulation (p-value < 0.05, ANOVA) (Fig 2A). This result suggests that A230V-ALK5 is a partial loss-of-function mutant that attenuates TGF-β1-mediated activation of a SMAD-reporter as compared to WT-ALK5. At a higher TGF-β1 concentration (5ng/mL), A230V-ALK5 and WT-ALK5 had similar relative luciferase activities (S2 Fig). To determine whether the results we observed in NIH/3T3 cells were reproducible in EC cells, we performed the previously described luciferase assays in the HEC-265 EC cell line (Fig 2B). HEC-265 cells were chosen because, similar to the EC tumor that harbored the endogenous A230V-ALK5 mutation, they are MSI+ (inferred) and have a similar mutation status across nine endometrial cancer genes (S2 Table). Similar to our findings in NIH/3T3 cells, expression of the A230V-ALK5 mutant in HEC-265 EC cells resulted in lower relative luciferase activity as compared to WT-ALK5 but higher luciferase activity as compared to the kinase-dead K232R-ALK5 control mutant, following stimulation with a high concentration of TGF-β1 (Fig 2B). However, the partial loss-of-function effect detected for A230V-ALK5 in HEC-265 transfected cells reached statistical significance only at the lowest TGF-β1 concentration, unlike in NIH/3T3 cells.

### The *A230V-ALK5* mutation encodes an unstable protein that undergoes increased ubiquitin-dependent degradation as compared to *WT-ALK5*

**Fig 3:**
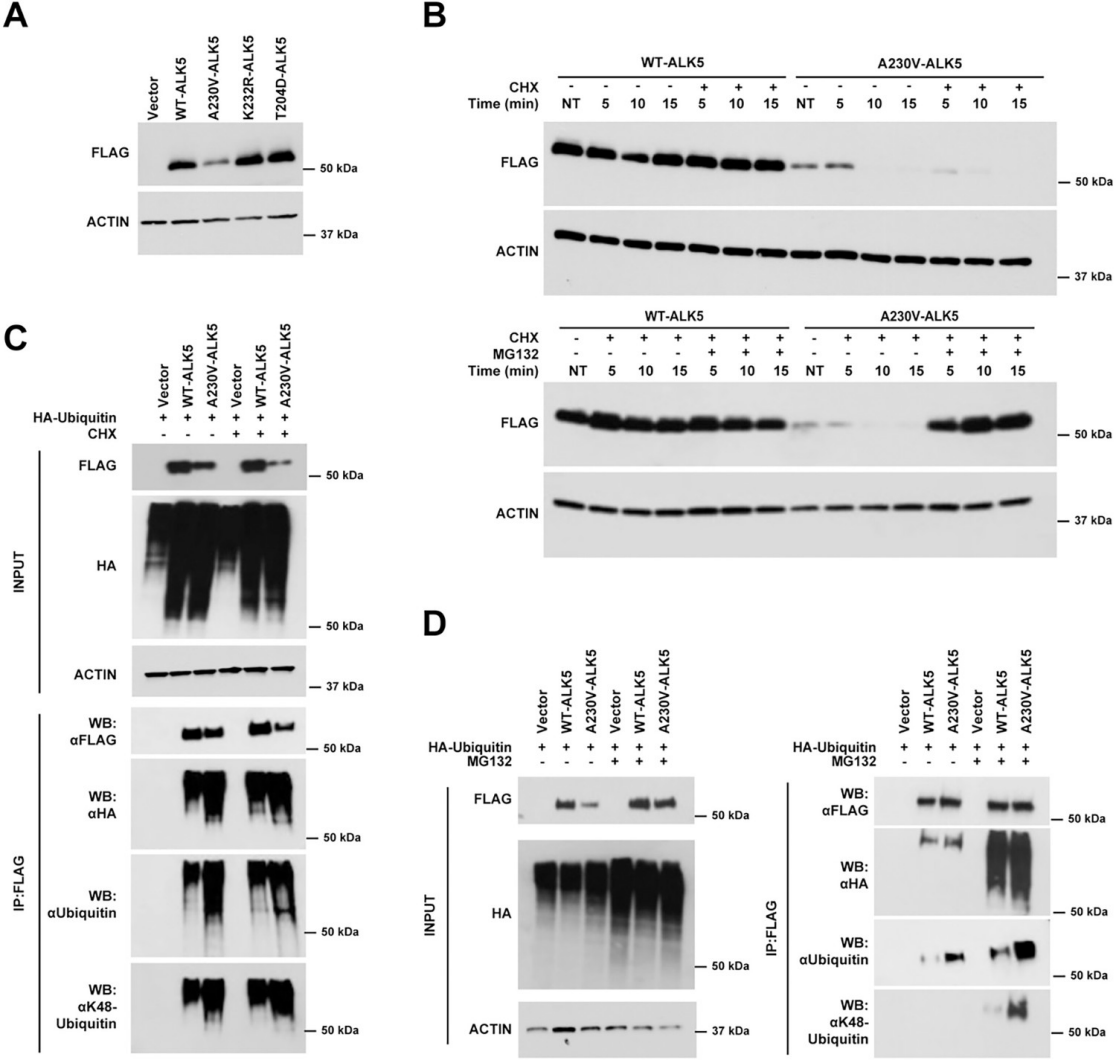
The A230V-ALK5 mutant is expressed at reduced levels compared with WT-ALK5 and undergoes rapid protein degradation. (A) NIH/3T3 cells were transiently transfected with FLAG-tagged ALK5 expression constructs and FLAG protein expression levels were measured by immunoblotting. (B) Transiently transfected NIH/3T3 cells were treated with vehicle or cycloheximide +/- MG132 and FLAG-ALK5 levels were measured by immunoblotting. (C, D) FLAG-ALK5 and HA-Ubiquitin constructs were co-transfected into NIH/3T3 cells followed by co-immunoprecipitation. The immune complexes were used for immunoblotting and probed with the antibodies indicated.

To determine whether the observed differences in SMAD-dependent reporter gene activity between the mutant A230V and wild-type proteins could be due to variation in expression levels of each protein in transiently transfected NIH/3T3 cells, protein lysates were subjected to immunoblotting using anti-FLAG monoclonal antibody (Fig 3A). Exogenously expressed FLAG-ALK5 was detected at variable levels between WT- and A230V-ALK5 transfected NIH/3T3 cells (Fig 3A). Specifically, A230V-ALK5 transfected cells consistently expressed lower steady-state levels of the exogenous FLAG-tagged protein as compared to WT-ALK5 transfected cells. The exogenous expression of A230V-ALK5 did not induce any observable morphologic changes over a 24-hour period (S4 Fig). We next sought to determine whether the reduced expression levels of FLAG-A230V-ALK5 were due to an increased rate of receptor degradation. We treated transfected cells with cycloheximide to inhibit protein synthesis and subsequently measured FLAG-ALK5 expression levels at 5-minute intervals over a 15-minute time-course. Results of this experiment showed a more rapid decrease in the steady-state levels of the FLAG-A230V-ALK5 protein than of the FLAG-WT-ALK5 protein (Fig 3B, top).

We next asked whether increased turnover by the ubiquitin-mediated proteasomal degradation pathway contributes to the shorter half-life of the A230V-ALK5 protein. NIH/3T3 cells were transiently transfected with either FLAG-tagged WT- or A230V-ALK5 constructs and treated with either DMSO or MG132 for 3 h followed by cycloheximide treatment for 15 min. The addition of MG132 to CHX resulted in stabilization of steady-state levels of A230V-ALK5 as compared to CHX-treatment alone (Fig 3B, bottom). The observed stabilization of A230V-ALK5 protein levels as a result of proteasome inhibitor treatment led us to speculate that the ubiquitin proteasome pathway was involved in regulating ALK5 protein turnover. Interestingly, despite the increase in levels of the A230V-ALK5 mutant protein following MG132 treatment, the relative luciferase activity of A230V-ALK5 upon TGF-β1 stimulation remained lower than that of the wildtype protein (S3 Fig).

Since proteasome inhibition results in the accumulation of polyubiquitinated proteins, we performed immunoprecipitation assays to compare ALK5 polyubiquitination levels in the presence of CHX, with or without MG132, following co-transfection of FLAG-tagged WT- or A230V-ALK5 expression constructs and an HA-tagged ubiquitin (Ub) expression construct into NIH/3T3 cells. Ubiquitin linkage-specific antibodies were used to distinguish K48- and K63-linked ubiquitin chains. Immunoprecipitation using an anti-FLAG antibody followed by immunoblotting using antibodies against ubiquitin revealed that ALK5 interacts with ubiquitin and undergoes polyubiquitination (Fig 3C). MG132 treatment in the presence of CHX stabilized both total ubiquitin and K48-linkage-specific ubiquitin levels for both WT- and A230V-ALK5 (Fig 3D). Cells transfected with A230V-ALK5 showed dramatically increased levels of polyubiquitin as detected by both anti-Ub and anti-K48-linkage specific ubiquitin antibodies, as compared to cells transfected with WT-ALK5 (Fig 3D). This result suggests that, compared to WT-ALK5, the A230V-ALK5 mutant is more prone to be polyubiquitinated and undergo ubiquitin-mediated proteasomal degradation. In summary, the lower steady-state level of the A230V-ALK5 mutant protein appears to be due, at least in part, to its increased ubiquitination and degradation via the proteasome, which might account for the reduced luciferase activity of this mutant in the reporter assays described above.

### The A230V-ALK5 mutant is associated with attenuated activation of canonical and non-canonical signaling pathways

ALK5 activates both canonical and noncanonical downstream signaling pathways. To determine whether the A230V-ALK5 mutation affects the canonical ALK5-SMAD2/3 signaling pathway, we first used immunoblotting to measure p-SMAD2(Ser465/Ser467) levels in NIH/3T3 cells transiently transfected with WT- or A230V-ALK5 constructs, in reduced-serum (2% calf serum) or full-serum (10% calf serum) culture conditions. In reduced-serum conditions, only the constitutively active T204D-ALK5 mutant control resulted in increased phosphorylation of p-SMAD2(Ser465/Ser467) as compared to the vector control (S5A Fig). In full-serum conditions, similar levels of p-SMAD2(Ser465/Ser467) were detected for WT-, A230V-mutant, and K232R-mutant-ALK5 transfectants (S5A Fig).

**Fig 4:**
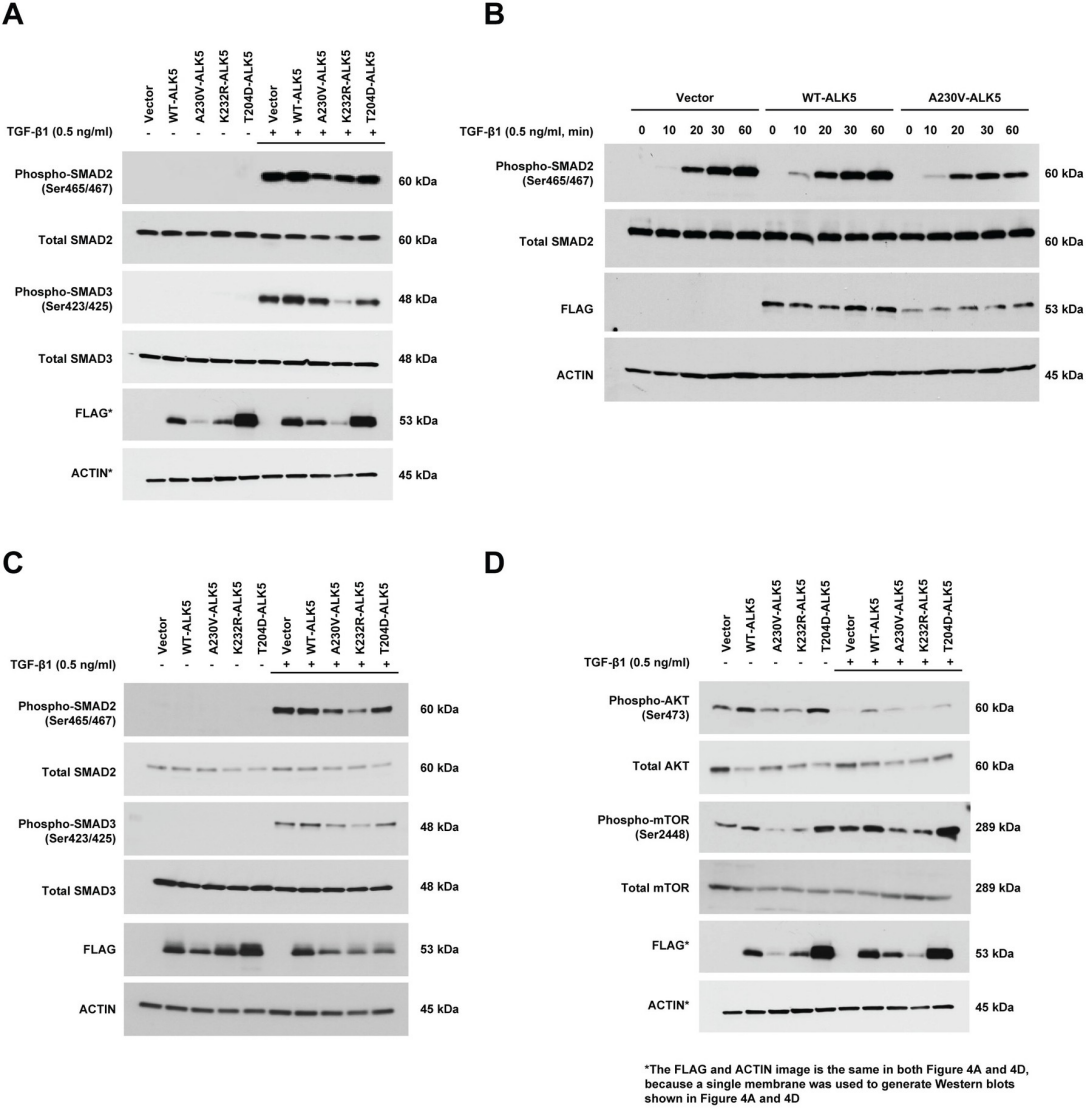
Transiently expressed A230V-ALK5 attenuates activation of downstream canonical and non-canonical signaling pathways in NIH/3T3 cells. (A) Immunoblots measuring levels of phosphorylated-SMAD2/3 in NIH/3T3 cells transiently transfected with control vector or the indicated FLAG-ALK5 expression constructs, before and after TGF-β1 (0.5ng/ml) stimulation for 30 min. A single membrane was used to generate Western blots shown in Fig 4A and 4D, so the actin image is the same in both Fig 4A and 4D. (B) Immunoblots measuring the effect of TGF-β1 (0.5ng/ml) stimulation for the indicated times (min) on phosphorylated-SMAD2 levels. (C) Immunoblots measuring levels of phosphorylated-SMAD2/3 in HEC-265 cells transiently transfected with control vector or the indicated FLAG-ALK5 expression constructs, before and after TGF-β1 (0.5ng/ml) stimulation. (D) Immunoblots measuring the effect of TGF-β1 (0.5ng/ml) stimulation on levels of total and phosphorylated forms of AKT and mTOR in NIH/3T3 cells transiently transfected with control vector or the indicated FLAG-ALK5 expression constructs. A single membrane was used to generate Western blots shown in Fig 4A and 4D, so the actin image is the same in both Fig 4A and 4D.

Next, we examined the effect of TGF-β1 ligand stimulation for 30 min on SMAD2 phosphorylation levels following serum-starvation (30 min) of transfected cells (Fig 4A). Our results showed that expression of the A230V-ALK5 mutant resulted in lower ligand-dependent p-SMAD2(Ser465/Ser467) levels as compared to WT-ALK5 or the vector-only control transfected cells. Expression of the K232R-ALK5 mutant resulted in lower ligand-induced p-SMAD2(Ser465/Ser467) and SMAD3(Ser423/Ser425) levels than expression of A230V-ALK5, WT-ALK5 or the vector-only control. Our finding that expression of the A230V-ALK5 mutant reduced basal p-SMAD2(Ser465/Ser467) levels below those of vector-only transfectants indicates that A230V-ALK5 might act in a dominant-negative manner.

Since the A230V-ALK5 mutant showed a partial reduction in activation of the canonical-SMAD2/3 pathway upon ligand stimulation, we next examined the temporal effect of ligand stimulation on the A230V-ALK5 mutant receptor by measuring p-SMAD2(Ser465/Ser467) levels in NIH/3T3 cells after 10, 20, 30, and 60 min of TGF-β1 ligand stimulation (Fig 4B). Our results showed that, compared to WT-ALK5 transfectants, p-SMAD2(Ser465/Ser467) levels were attenuated following 10 and 20 min of TGF-β1 stimulation in A230V-ALK5 transfectants (Fig 4B). We speculate that the attenuated activation of SMAD2 by A230V-ALK5 may be due to the reduced protein stability of this mutant based on our *in vitro* data (above).

We also evaluated TGF-β1-induced canonical SMAD2/3 signaling in HEC-265 EC cells following transient transfection with ALK5 constructs. Similar to our findings in NIH/3T3 cells, we found that in HEC-265 cells canonical SMAD2 and SMAD3 activation was ligand-dependent and phosphorylation levels of SMAD2 and SMAD3 were lower in A230V-ALK5 transfectants compared with WT-ALK5 transfectants (Fig 4C).

One non-canonical signaling pathway regulated by ALK5 is the AKT-mTOR pathway. In contrast with endogenous SMAD2/3 signaling in HEC-265 cells, endogenous mTOR and AKT were constitutively activated in HEC-265 EC cells (S5 Fig, Fig 4B). Therefore, to investigate whether the A230V-ALK5 mutation alters AKT-mTOR signaling, we measured p-AKT(Ser473) and p-mTOR(Ser2448) levels in ALK5-transfected NIH/3T3 cells, in the presence and absence of TGF-β ligand stimulation (Fig 4D). We found that in NIH/3T3 cells exogenous expression of the A230V-ALK5 mutant resulted in lower levels of both p-AKT(Ser473) and p-mTOR(Ser2448) before and after TGF-β1 ligand treatment as compared to WT-ALK5 (Fig 4D). This result indicates that compared to WT-ALK5 the A230V-mutant acts as a partial loss of function mutant in response to both cellular stress (i.e. serum-starved conditions) and to TGF-β ligand stimulation in NIH/3T3 cells.

### Activity of the A230V-ALK5 mutant is inhibited less by SB-435142 or galunisertib than that of WT-ALK5

**Fig 5:**
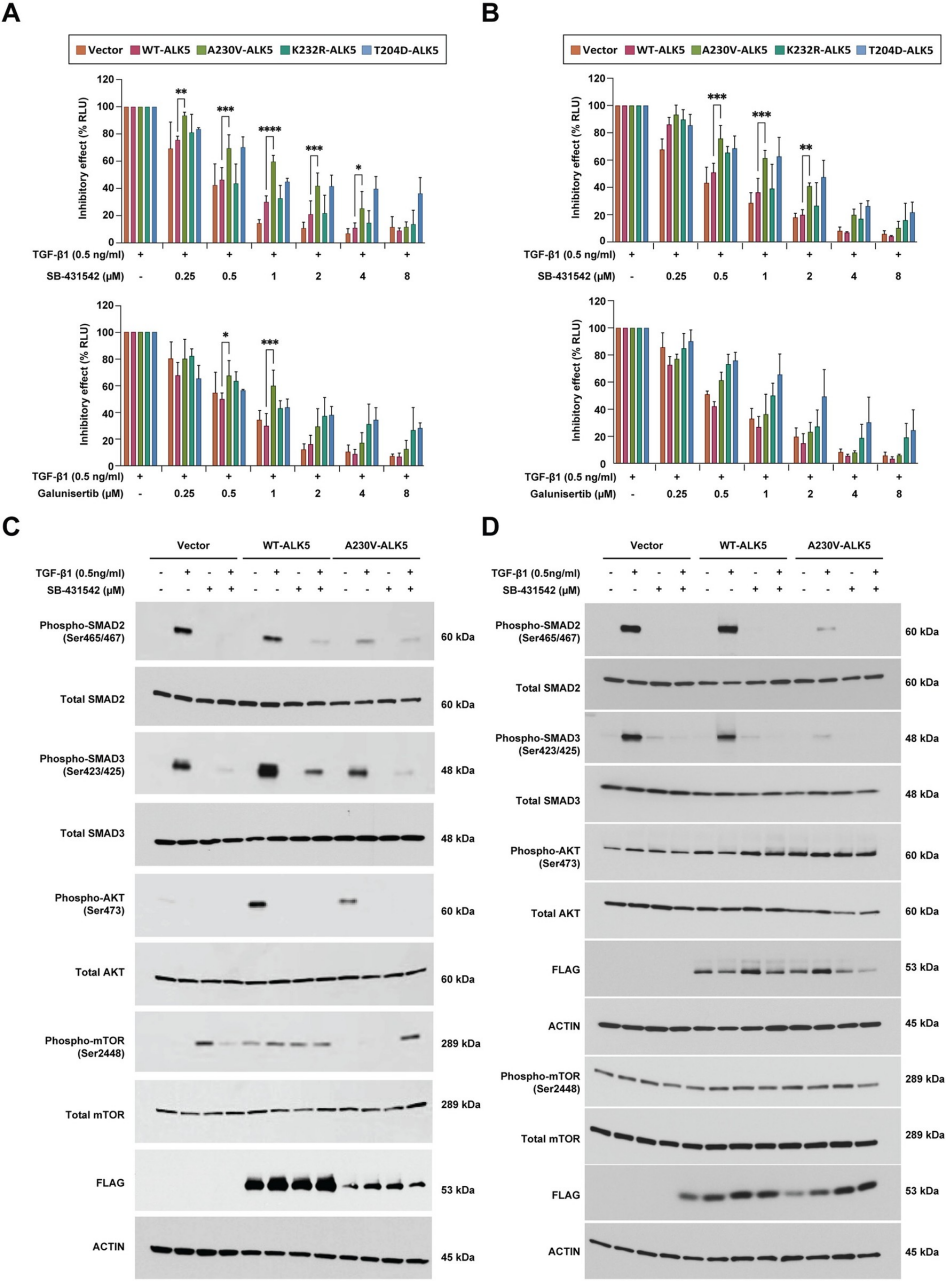
A230V-ALK5 activity shows reduced sensitivity to inhibition by SB-435412 and galunisertib, as compared to WT-ALK5. SMAD binding element (SBE) firefly luciferase reporter assays in (A) NIH/3T3 cells and (B) HEC-265 EC cells. Immunoblots measuring levels of total and phosphorylated forms of SMAD2, SMAD3, AKT, and mTOR in (C) NIH/3T3 cells and (D) HEC-265 EC cells, in the presence or absence of TGF-β1 stimulation and/or SB-431542 treatment.

Our structural modeling predicts that SB-431542, an ATP-competitive small molecule inhibitor of ALK5, has reduced affinity to bind to the A230V-ALK5 mutant as compared to WT-ALK5. We therefore examined the effects of SB-431542 and galunisertib, another ALK5 ATP-competitive small molecule inhibitor, on WT- and mutant-ALK5 activity in both NIH/3T3 cells and HEC-265 EC cells. In SBE luciferase reporter assays (Fig 5A and 5B), our results showed that the TGF-β-induced luciferase reporter activity of A230V-ALK5 transfected cells was less sensitive to inhibition by SB-431542 or galunisertib than WT-ALK5 transfected cells, particularly across the lower ranges of drug concentrations tested.

These results were obtained for both NIH/3T3 (Fig 5A) and HEC-265 (Fig 5B). For SB-431542, the half-maximal inhibitory concentration (IC_50_) for A230V-ALK5 transfected NIH/3T3 cells was 3.27-fold higher than the IC_50_ for WT-ALK5 transfected cells (1.44 mM *versus* 0.44 mM, respectively) (S6A Fig). This result indicates that the A230V-ALK5 mutant is less sensitive to inhibition by SB-431542 than WT-ALK5, potentially due to the predicted changes in binding affinity of the A230V mutant to this inhibitor, as noted above. Similarly, galunisertib showed a 2.12-fold higher IC_50_ for A230V-ALK5 transfected NIH/3T3 cells as compared to WT-ALK5 transfected cells (1.02 mM *versus* 0.48 mM, respectively). In HEC-265 cells, we observed that the TGF-β-induced luciferase reporter activity of the A230V-ALK5 mutant also displayed significantly less inhibition by SB-431542 and galunisertib, especially at lower drug concentrations (Fig 5B). Similar to our results in NIH/3T3 cells, in HEC-265 cells IC_50_ values were 2.4-fold higher (0.36 mM *versus* 0.15 mM) for SB-431542 and 1.7-fold higher (0.17 mM *versus* 0.1 mM) for galunisertib in A230V-ALK5 transfectants than in WT-ALK5 transfectants (S6B Fig).

Previous studies have shown that SB-431542 is a potent inhibitor of ALK5 and inhibits SMAD2 phosphorylation. We next tested which signaling pathway is affected by small molecule inhibition of the A230V-ALK5 mutant in both NIH/3T3 cells and HEC-265 EC cells (Fig 5C and 5D). Protein lysates for immunoblotting were collected after pretreatment with SB-431542 for 1h followed by TGF-β1 stimulation for 30min. We examined canonical SMAD2/3 signaling and non-canonical PI3K/AKT signaling, based on our earlier finding of reduced AKT signaling in A230V-ALK5 NIH/3T3 transfectants. Consistent with results of our luciferase assays, levels of ligand-dependent p-SMAD2(Ser465/467) and p-SMAD3(Ser423/425) in the A230V-ALK5 transfectants were less inhibited by SB-431542 than in the WT-ALK5 transfectants. Ligand-independent phospho-AKT levels were inhibited by SB-431542 in WT- and A230V-ALK5 transfectants for NIH/3T3. Phospho-mTOR(Ser2448) levels were not affected by SB-431542 in WT-ALK5 or A230V-ALK5 transfectants in the NIH/3T3 cell line. In the HEC-265 EC cell line, phospho-AKT and phospho-mTOR levels were unaffected by SB-431542 in ALK5 transfected cells.

## Discussion

Protein kinases are activated upon ATP-binding and, as a consequence, phosphorylate other proteins to regulate downstream signaling cascades and relevant biological processes. Within the ALK5 ATP-binding pocket, the alanine residue at position 230 is conserved in 401 members of the protein kinase family, and has been annotated as a “significantly mutated position” in a pan-cancer analysis [30]. Moreover, residue A230 and nearby residues are being targeted and used for the design of ATP-competitive inhibitors, which makes it important to study the functional effects of naturally-occurring mutations at these residues. Herein, we show that the A230V-ALK5 endometrial cancer mutant acts as a partial loss-of-function mutant that has attenuated TGFβ-signaling and exhibits reduced biochemical inhibition by ALK5 inhibitors.

To begin to understand the functional consequences of the A230V-ALK5 mutation, we modeled the mutant protein using molecular docking algorithms, which predict the preferred orientation of a given ligand (ATP or inhibitor) within the binding pocket of a target protein and assist in evaluating the affinity of kinase-ligand complexes via electrostatic interactions and van der Waals interactions. The results of our molecular docking studies showed that a valine substitution at position 230 of ALK5 increases the distance between the binding pocket of the mutant protein and the ATP ligand. Based on this result, we speculate that ATP has reduced access to the catalytic cleft of the mutant A230V-ALK5 protein.

The results of our kinase assays demonstrate that the A230V-ALK5 mutation disrupts TGF-β-dependent kinase activity, particularly at lower TGF-β1 concentrations. Specifically, the A230V-ALK5 mutant exhibits attenuated kinase activity as compared to the K232R-ALK5 kinase-dead mutant. This effect is possibly due to the reduced access of ATP to the binding pocket of the A230V-ALK5 mutant protein, which we predicted from our molecular docking results. Our findings suggest that the wildtype A230 residue contributes to optimal ligand-dependent kinase activity but is not essential for ligand-dependent kinase activation.

In addition to the attenuated kinase activity of A230V-ALK5, our study also demonstrates that the mutant protein is less stable than wildtype-ALK5. We show that when transiently over-expressed in NIH/3T3 cells, newly synthesized FLAG-tagged ALK5 proteins were degraded in a time-dependent and proteasome-dependent manner, but the A230V mutant protein exhibited an increased rate of degradation compared to the wildtype protein. Consistent with this result, the A230V-ALK5 mutant was polyubiquitinated at a higher level than WT-ALK5 and showed an increase in K48-linkage specific ubiquitination. Interestingly, despite the increase in levels of the mutant protein following MG132 treatment, the kinase activity of A230V-ALK5 remained lower than that of the wildtype protein (S3 Fig). Collectively, our results show that the A230V mutation in ALK5 is closely associated with both altered kinase activity and altered protein stability. To our knowledge, this is the first report of reduced stability of a mutant ALK5 protein due to enhanced proteasomal-mediated degradation.

We also show by immunoblotting that the A230V-ALK5 mutant results in impaired canonical SMAD2/3 signaling. Our time-course measuring phosphorylated SMAD2 levels showed an attenuated response to TGF-β stimulation by the A230V mutant. This effect might be due to the reduced stability of the mutant as reported herein. TGF-β activation of ALK5 can also result in the activation of non-canonical SMAD-independent pathways, including the PI3K/AKT/mTOR pathway. Here we showed that the A230V mutant was associated with reduced levels of phosphorylated AKT and mTOR following TGF-β stimulation. This effect is consistent with the reduced level of phospho-AKT associated with the pathogenic germline A230T-ALK5 Loeys-Dietz syndrome mutant, as reported by others [31]. In contrast, non-canonical AKT/mTOR signaling in HEC-265 EC cells exhibited high basal levels, without ligand stimulation, and no significant differences were detected in AKT/mTOR signaling between WT-ALK5 and A230V-ALK5 transfectants either with or without ligand stimulation.

We extended our study to determine the biochemical sensitivity of the A230V-ALK5 mutant to ALK5 ATP competitive inhibitors, relative to WT-ALK5. Therapeutic strategies targeting ALK5 have been considered particularly in the context of diseases characterized by excessive fibrosis, such as idiopathic pulmonary fibrosis, liver fibrosis, cardiovascular disease, and certain cancers [32–34]. Small molecules such as SB-431542 and LY2157299 (galunisertib), as well as monoclonal antibodies that specifically inhibit ALK5 signaling, can block receptor activation and prevent downstream signaling responsible for fibroblast activation and extracellular matrix deposition [34]. Clinical trials to assess the efficacy of combining ALK5 inhibitors in conjunction with other therapeutics, such as PD-1/PD-L1 immune checkpoint inhibitors or anti-inflammatory drugs, are also underway [35–37]. Additionally, strategies utilizing RNA interference (e.g., siRNA) to reduce ALK5 expression or its signaling components are under investigation [32]. However, challenges remain, particularly in selectively targeting ALK5 without affecting other TGF-β receptor family members to minimize side effects. Furthermore, tumors may develop acquired resistance to ALK5 inhibitors, highlighting the need for further research into combination strategies or novel agents.

We compared the kinase activity of WT-ALK5 and A230V-ALK5 transfected NIH/3T3 cells and HEC-265 cells, following treatment with SB-435142 (an ALK5/4/7 inhibitor) or with galunisertib (an ALK5 inhibitor). Both agents successfully inhibited ALK5 kinase activity, but the activity of the A230V mutant was inhibited to a lesser extent than that of WT-ALK5. Consistent with this experimental finding, our ligand docking simulation result predicts the A230V protein has a reduced binding affinity to the inhibitor and higher Ki value than WT-ALK5, possibly due to the conformational change in the ATP binding pocket. Consistent with our ligand stimulation data, both SMAD2 and SMAD3 phosphorylation levels were attenuated by the A230V mutant, as were non-canonical PI3K and mTOR signaling with ALK5 inhibitor treatment, but changes in AKT phosphorylation levels were not observed for WT nor the A230V mutant. The molecular mechanisms underlying the reduced inhibitor sensitivity of A230V-ALK5 to small molecule inhibitors will require further exploration.

In summary, our functional characterization of the A230V missense mutant in the ATP binding pocket of ALK5 shows that it is a partial loss-of-function mutation that disrupts TGFβ-induced signaling pathways. In addition, newly synthesized A230V mutant protein exhibits an increased rate of degradation via the ubiquitin-proteasome pathway. Further investigations into the functional effects of the A230V-ALK5 mutant on downstream non-canonical signaling pathways and the signaling pathways that may mediate the sensitivities of the A230V mutant proteins to ATP-competitive inhibitors are warranted.

## Supporting information

S1 Raw images Raw images underlying all western blot results. (PDF)

S2 Raw images Additional replicates underlying all western blot results. (PDF)

S1 File Raw PDB files for A230V-ALK5 AutoDock Vina docking with ATP orientation 1. (PSE)

S2 File Raw PDB files for A230V-ALK5 AutoDock Vina docking with ATP orientation 2. (PSE)

S3 File Raw PDB files for wildtype-ALK5 AutoDock Vina docking with ATP orientation 1. (PSE)

S4 File Raw PDB files for wildtype-ALK5 AutoDock Vina docking with ATP orientation 2. (PSE)

S5 File Raw PDB files for A230V-ALK5 doc AutoDock Vina docking with SB-43152. (PSE)

S6 File Raw PDB files for wildtype-ALK5 AutoDock Vina docking with SB-43152. (PSE)

S1 Fig Spectrum of ALK5 somatic mutations in non-ultramutated endometrial tumors in The Cancer Genome Atlas (TCGA) (https://www.cancer.gov/tcga). Lollipop plot indicates the positions of somatic mutations relative to ALK5 functional domains. Each circle (red) represents a single mutation; the ALK5-R255C kinase domain mutant is recurrent. Abbreviations: GS, glycine (G) and serine (S) rich sequence.
(TIF)

S2 Fig The A230V-ALK5 mutant behaves like WT-ALK5 in luciferase reporter assays following TGF-β1 stimulation at the highest concentration (5 ng/mL) tested. Results of TGF-β1-responsive SMAD binding element (SBE) firefly luciferase reporter assays in NIH/3T3 cells. Cells were triply transfected with pGL4.48[SBE] Firefly luciferase reporter construct, pGL4.74[TK] Renilla vector and either a wildtype (WT) or mutant FLAG-tagged ALK5 expression construct or control vector (CV). Expression constructs containing the ALK5-T204D (constitutively active mutant) or -K232R (kinase-dead) mutant were included as positive and negative controls. After 24 h transfection, cells were serum-starved for 30 min before TGF-β1 was added at the indicated concentration for an additional 3 h and luciferase activity was measured after the addition of assay reagents based on manufacturer’s protocol (Dual-Glo luciferase assay system). Relative Luciferase Units were calculated by normalizing SBE luciferase activity to Renilla luciferase activity. The mean ± SD values, based on results of four independent experiments, were calculated using Prism software (GraphPad, Boston, MA) and plotted. P-values were calculated using Two-Way ANOVA; *P<0.05.
(TIF)

S3 Fig The A230V-ALK5 mutant behaves as a partial loss-of-function mutant in luciferase reporter assays following TGF-β1 stimulation and MG132 treatment. Results of TGF-β1-responsive SMAD binding element (SBE) firefly luciferase reporter assays in NIH/3T3 cells, following MG132 treatment. Cells were triply transfected with pGL4.48[SBE] Firefly luciferase reporter construct, pGL4.74[TK] Renilla vector and either a wildtype (WT) or mutant FLAG-tagged ALK5 expression construct or control vector (CV). Expression constructs containing the ALK5-T204D (constitutively-active mutant) or -K232R (kinase-dead) mutants were included as positive and negative controls. After 24 h transfection, cells were serum-starved for 30 min before treatment with MG132 for 1 h, followed by TGF-β1 treatment for 3 h. RLU was measured using Dual-Glo luciferase assay system and calculated by normalizing SBE luciferase activity to Renilla luciferase activity. The mean ± SD, based on results of four independent experiments were calculated using Prism software (GraphPad, Boston, MA) and plotted. P-values were calculated using Two-Way ANOVA; *P<0.05.
(TIF)

S4 Fig The A230V-ALK5 mutant did not change cell morphology after 24 h transfection. NIH/3T3 cells were transiently transfected with FLAG-tagged ALK5 expression constructs. There were no visible differences in cell morphology 24 h after transfection between (A) WT and (B) A230V mutant cells (10X magnification).
(TIF)

S5 Fig (A) Immunoblots measuring levels of phosphorylated-SMAD2 in NIH/3T3 cells transiently transfected with control vector or the indicated FLAG-ALK5 expression constructs, in serum (2%)-deprived culture medium (left) or complete (10%) serum (right). (B) Immunoblots measuring levels of phosphorylated-AKT and phosphorylated-mTOR in HEEC-265 cells transiently transfected with control vector or the indicated FLAG-ALK5 expression constructs.
(TIF)

S6 Fig IC_50_ values for the ALK5 inhibitors SB-431542 and Galunisertib based on luciferase reporter assays conducted in (A) NIH/3T3 cells and (B) HEC-265 cells transfected with the indicated ALK5 constructs and treated with TGF-β1. Nonlinear regression curve fitting was performed using Prism software to plot each graph from four independent experiments. RLU: relative luciferase units.
(TIF)

S7 Fig A graphical abstract of the study. (Top) Molecular docking with ALK5 protein with their ligand, ATP, and SB-431542. (Bottom) In vitro experiment results show the A230V-ALK5 mutant cells show a decrease in both canonical SMAD2/3 and non-canonical signaling and inhibitor sensitivity but show an increase in proteasomal degradation of the receptor protein.
(TIF)

S1 Table Interaction, van der Waals, electrostatic and hydrogen bond energies of protein-ligand complex calculated by Autodock Vina. (DOCX)

S2 Table MSI status and mutation status of nine EC-associated genes, and of *TGFBR2*, in the ALK5-A230V-mutated endometrial tumor and the HEC-265 EC cell line. (DOCX)

## References

[1] Derynck R, Budi EH. Specificity, versatility, and control of TGF-β family signaling. Sci Signal. 2019;12:eaav5183. 10.1126/scisignal.aav5183 PMC6800142 30808818

[2] Peng J, Monsivais D, You R, Zhong H, Pangas SA, Matzuk MM. Uterine activin receptor-like kinase 5 is crucial for blastocyst implantation and placental development. Proc Natl Acad Sci U S A. 2015;112:E5098–107. doi: 10.1073/pnas.1514498112 26305969 PMC4568667

[3] Ni N, Li Q. TGFβ superfamily signaling and uterine decidualization. Reprod Biol Endocrinol. 2017;15:84. 29029620 10.1186/s12958-017-0303-0 PMC5640934

[4] Fang X, Ni N, Gao Y, Lydon JP, Ivanov I, Rijnkels M, et al. Transforming growth factor beta signaling and decidual integrity in mice†. Biol Reprod. 2020;103:1186–1198. doi: 10.1093/biolre/ioaa155 32902612 PMC7711917

[5] Monsivais D, Peng J, Kang Y, Matzuk MM. Activin-like kinase 5 (ALK5) inactivation in the mouse uterus results in metastatic endometrial carcinoma. Proc Natl Acad Sci U S A. 2019;116:3883–3892. 30655341 10.1073/pnas.1806838116 PMC6397539

[6] Gao Y, Lin P, Lydon JP, Li Q. Conditional abrogation of transforming growth factor-β receptor 1 in PTEN-inactivated endometrium promotes endometrial cancer progression in mice. J Pathol. 2017;243:89–99. 28657664 10.1002/path.4930 PMC5568928

[7] Cancer Facts & Figures 2024. Atlanta: American Cancer Society, Inc. 2024. Available from: https://www.cancer.org/research/cancer-facts-statistics/all-cancer-facts-figures/2024-cancer-facts-figures.html

[8] Rahib L, Wehner MR, Matrisian LM, Nead KT. Estimated Projection of US Cancer Incidence and Death to 2040. JAMA Netw Open. 2021;4:e214708. doi: 10.1001/jamanetworkopen.2021.4708 33825840 PMC8027914

[9] Korkut A, Zaidi S, Kanchi RS, Rao S, Gough NR, Schultz A, et al. A Pan-Cancer Analysis Reveals High-Frequency Genetic Alterations in Mediators of Signaling by the TGF-β Superfamily. Cell Syst. 2018;7:422–437. 30268436 10.1016/j.cels.2018.08.010 PMC6370347

[10] Teicher BA. TGFβ-Directed Therapeutics: 2020. Pharmacol Ther. 2021;217:107666. 32835827 10.1016/j.pharmthera.2020.107666 PMC7770020

[11] Cerami E, Gao J, Dogrusoz U, Gross BE, Sumer SO, Aksoy BA, et al. The cBio cancer genomics portal: an open platform for exploring multidimensional cancer genomics data. Cancer Discov. 2012;2:401–4. doi: 10.1158/2159-8290.CD-12-0095 22588877 PMC3956037

[12] Gao J, Aksoy BA, Dogrusoz U, Dresdner G, Gross B, Sumer SO, et al. Integrative analysis of complex cancer genomics and clinical profiles using the cBioPortal. Sci Signal. 2013;6:pl1. doi: 10.1126/scisignal.2004088 23550210 PMC4160307

[13] Berger AC, Korkut A, Kanchi RS, Hegde AM, Lenoir W, Liu W, et al. A Comprehensive Pan-Cancer Molecular Study of Gynecologic and Breast Cancers. Cancer Cell. 2018;33:690–705. doi: 10.1016/j.ccell.2018.03.014 29622464 PMC5959730

[14] Soumerai TE, Donoghue MTA, Bandlamudi C, Srinivasan P, Chang MT, Zamarin D, et al. Clinical Utility of Prospective Molecular Characterization in Advanced Endometrial Cancer. Clin Cancer Res. 2018;24:5939–5947. doi: 10.1158/1078-0432.CCR-18-0412 30068706 PMC6279519

[15] Manning-Geist BL, Liu YL, Devereaux KA, Paula ADC, Zhou QC, Ma W, et al. Microsatellite Instability-High Endometrial Cancers with MLH1 Promoter Hypermethylation Have Distinct Molecular and Clinical Profiles. Clin Cancer Res. 2022;28:4302–4311. doi: 10.1158/1078-0432.CCR-22-0713 35849120 PMC9529954

[16] Cherniack AD, Shen H, Walter V, Stewart C, Murray BA, Bowlby R, et al. Integrated Molecular Characterization of Uterine Carcinosarcoma. Cancer Cell. 2017;31:411–423. doi: 10.1016/j.ccell.2017.02.010 28292439 PMC5599133

[17] Ng PC, Henikoff S. SIFT: Predicting amino acid changes that affect protein function. Nucleic Acids Res. 2003;31:3812–4. doi: 10.1093/nar/gkg509 12824425 PMC168916

[18] Reva B, Antipin Y, Sander C. Predicting the functional impact of protein mutations: application to cancer genomics. Nucleic Acids Res. 2011;39(17):e118. doi: 10.1093/nar/gkr407 21727090 PMC3177186

[19] Adzhubei IA, Schmidt S, Peshkin L, Ramensky VE, Gerasimova A, Bork P, et al. A method and server for predicting damaging missense mutations. Nat Methods. 2010;7:248–249. doi: 10.1038/nmeth0410-248 20354512 PMC2855889

[20] López-Ferrando V, Gazzo A, Cruz X, Orozco M, Gelpí J. PMut: a web-based tool for the annotation of pathological variants on proteins, 2017 update. Nucleic Acids Res. 2017;45: W222–W228. doi: 10.1093/nar/gkx313 28453649 PMC5793831

[21] Hecht M, Bromberg Y, Rost B. Better prediction of functional effects for sequence variants. BMC Genomics. 2015;16(Suppl 8):S1. doi: 10.1186/1471-2164-16-S8-S1 26110438 PMC4480835

[22] Thomas PD, Ebert D, Muruganujan A, Mushayahama T, Albou LP, Mi H. PANTHER: Making genome-scale phylogenetics accessible to all. Protein Sci. 2022;31:8–22. doi: 10.1002/pro.4218 34717010 PMC8740835

[23] Ioannidis NM, Rothstein JH, Pejaver V, Middha S, McDonnell SK, Baheti S, et al. REVEL: An Ensemble Method for Predicting the Pathogenicity of Rare Missense Variants. Am J Hum Genet. 2016;99:877–885. doi: 10.1016/j.ajhg.2016.08.016 27666373 PMC5065685

[24] Eberhardt J, Santos-Martins D, Tillack A, and Forli S. AutoDock Vina 1.2.0: New Docking Methods, Expanded Force Field, and Python Bindings. J. Chem. Inf. Model. 2021, 61, 8, 3891–3898. doi: 10.1021/acs.jcim.1c00203 34278794 PMC10683950

[25] Trott O, Olson AJ. AutoDock Vina: improving the speed and accuracy of docking with a new scoring function, efficient optimization, and multithreading. J Comput Chem. 2010;31:455–61 doi: 10.1002/jcc.21334 19499576 PMC3041641

[26] Forli S, Huey R, Pique M, Sanner M, Goodsell D, Olson A. Computational protein–ligand docking and virtual drug screening with the AutoDock suite. Nat Protoc 2016;11:905–919. doi: 10.1038/nprot.2016.051 27077332 PMC4868550

[27] Fujisawa T, Hamano M, Hata H, Kamata Y, Watanabe J, Sekimoto R, et al. Establishment and characterization of two different types of new human endometrial adenocarcinoma cell lines (HEC-251 and HEC-265). Eur J Gynaecol Oncol. 2004;25:299–304. 15171304

[28] Ogunjimi AA, Zeqiraj E, Ceccarelli DF, Sicheri F, Wrana JL, David L. Structural basis for specificity of TGFβ family receptor small molecule inhibitors. Cell Signal. 2012;24:476–483. 21983015 10.1016/j.cellsig.2011.09.027 PMC4490768

[29] Moren A, Imamura T, Miyazono K, Heldin CH, Moustakas A. Degradation of the tumor suppressor Smad4 by WW and HECT domain ubiquitin ligases. J Biol Chem. 2005;280:22115–23. doi: 10.1074/jbc.M414027200 15817471

[30] Kumar RD, Bose R. Analysis of somatic mutations across the kinome reveals loss-of-function mutations in multiple cancer types. Sci Rep. 2017;7:6418. doi: 10.1038/s41598-017-06366-x 28743916 PMC5527104

[31] Zhou D, Feng H, Yang Y, Huang T, Qiu P, Zhang C, et al. hiPSC Modeling of Lineage-Specific Smooth Muscle Cell Defects Caused by *TGFBR1*^A230T^ Variant, and Its Therapeutic Implications for Loeys-Dietz Syndrome. Circulation. 2021;144:1145–1159. 34346740 10.1161/CIRCULATIONAHA.121.054744 PMC8681699

[32] Yingling J, Blanchard K, Sawyer J. Development of TGF-beta signaling inhibitors for cancer therapy. Nat Rev Drug Discov. 2004;3(12):1011–1022. 15573100 10.1038/nrd1580

[33] Neuzillet C, Tijeras-Raballand A, Cohen R, Cros J, Faivre S, Raymond E, et al. Targeting the TGFβ pathway for cancer therapy. Pharmacol Ther. 2015;147:22–31. 25444759 10.1016/j.pharmthera.2014.11.001

[34] Akhurst R, Hata A. Targeting the TGFβ signalling pathway in disease. Nat Rev Drug Discov. 2012;11(10):790. 23000686 10.1038/nrd3810 PMC3520610

[35] Gulley J, Schlom J, Barcellos-Hoff M, Wang X, Seoane J, Audhuy F, et al. Dual inhibition of TGF-β and PD-L1: a novel approach to cancer treatment. Mol Oncology. 2022;16:2117–2134. 10.1002/1878-0261.13146 PMC9168966 34854206

[36] Lind H, Gameiro S, Jochems C, Donahue R, Strauss J, Gulley J, et al. Dual targeting of TGF-β and PD-L1 via a bifunctional anti-PD-L1/TGF-βRII agent: status of preclinical and clinical advances. J Immunother Cancer. 2020;8:e000433:e1426519. 32079617 10.1136/jitc-2019-000433 PMC7057416

[37] Cheng B, Ding K, Chen P, Ji J, Luo T, Guo X, et al. Anti-PD-L1/TGF-βR fusion protein (SHR-1701) overcomes disrupted lymphocyte recovery-induced resistance to PD-1/PD-L1 inhibitors in lung cancer. Cancer Commun (Lond). 2022;42:17–36. 34981670 10.1002/cac2.12244 PMC8753312

