# Supplementary material for: The endometrial cancer A230V-ALK5 (TGFBR1) mutant attenuates TGF-β signaling and exhibits reduced in vitro sensitivity to ALK5 inhibitors
Source: PLoS One. 2024 Nov 22;19(11):e0312806. doi: 10.1371/journal.pone.0312806 (PMC11584080; doi:10.1371/journal.pone.0312806)

Figure 3A- Extended image

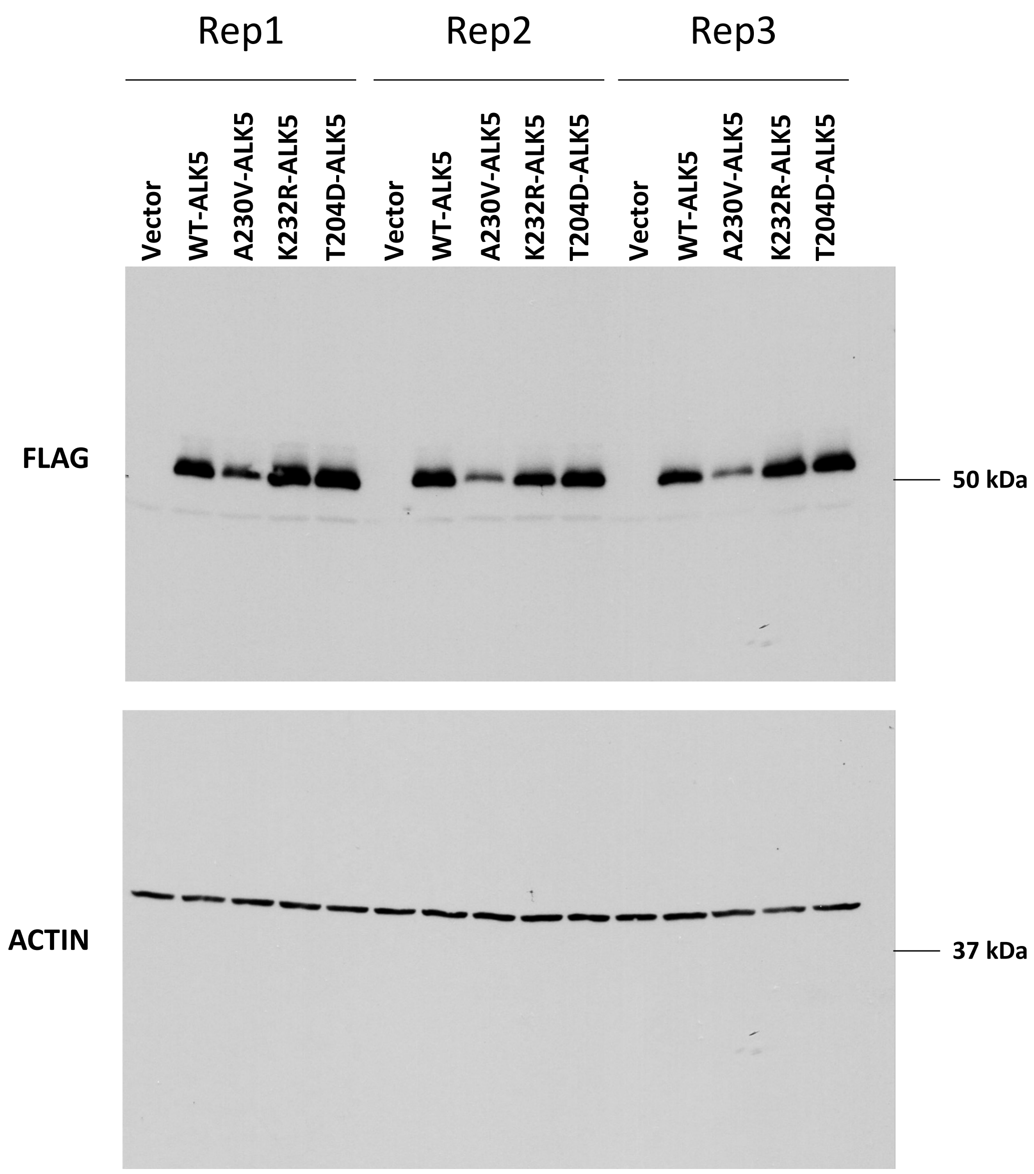

Figure 3B- extended image

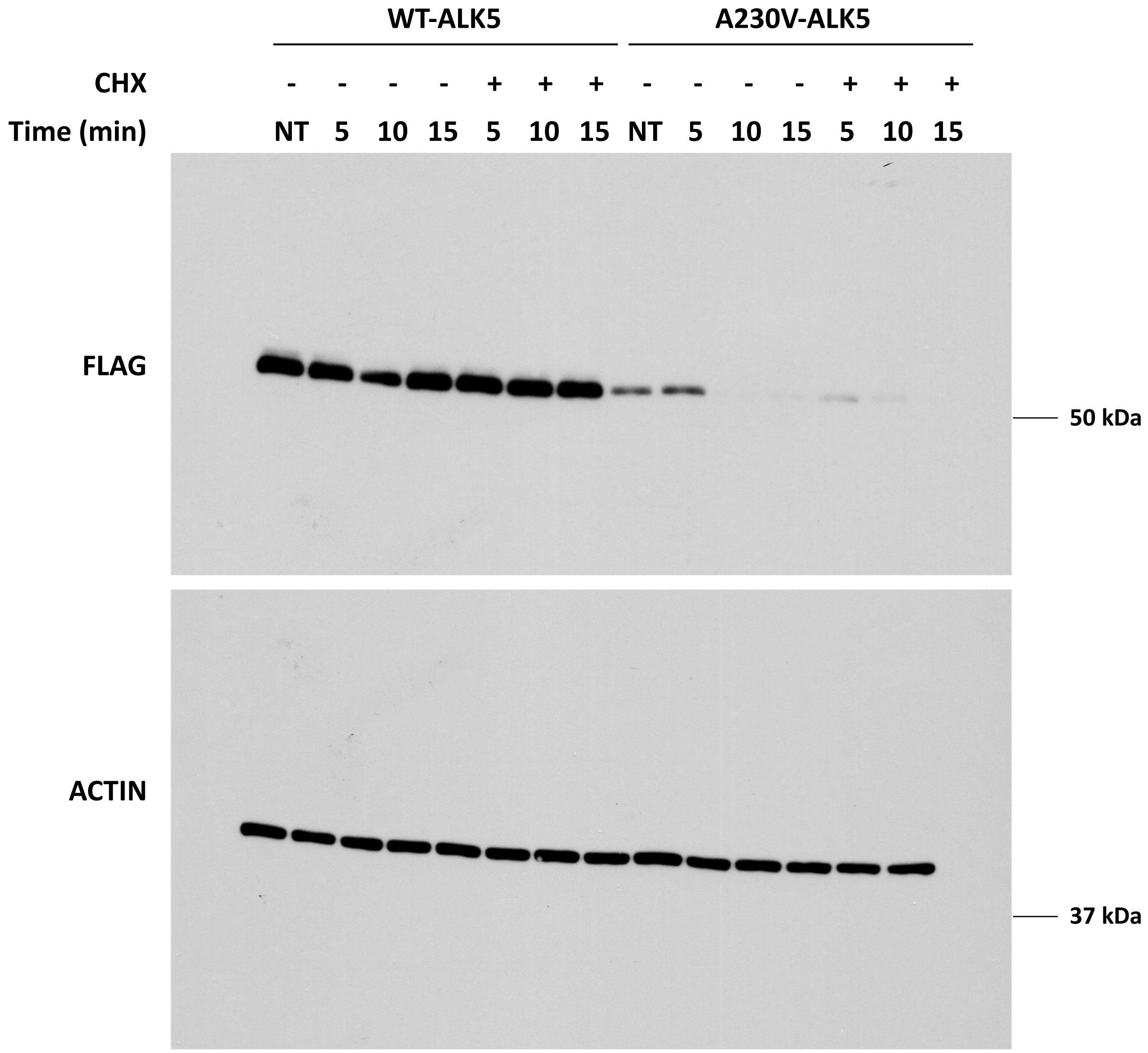

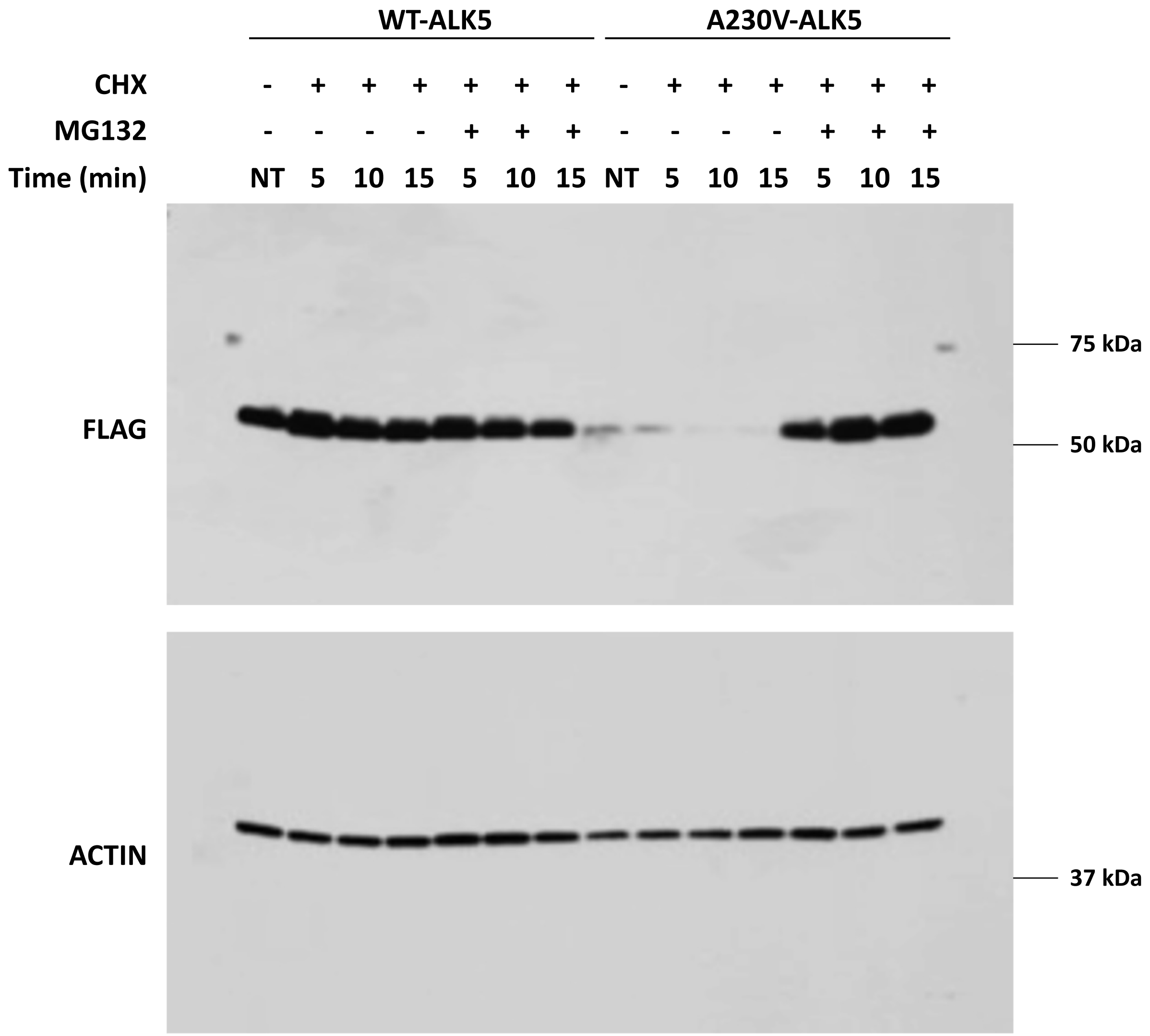

Figure 3C, D- extended image

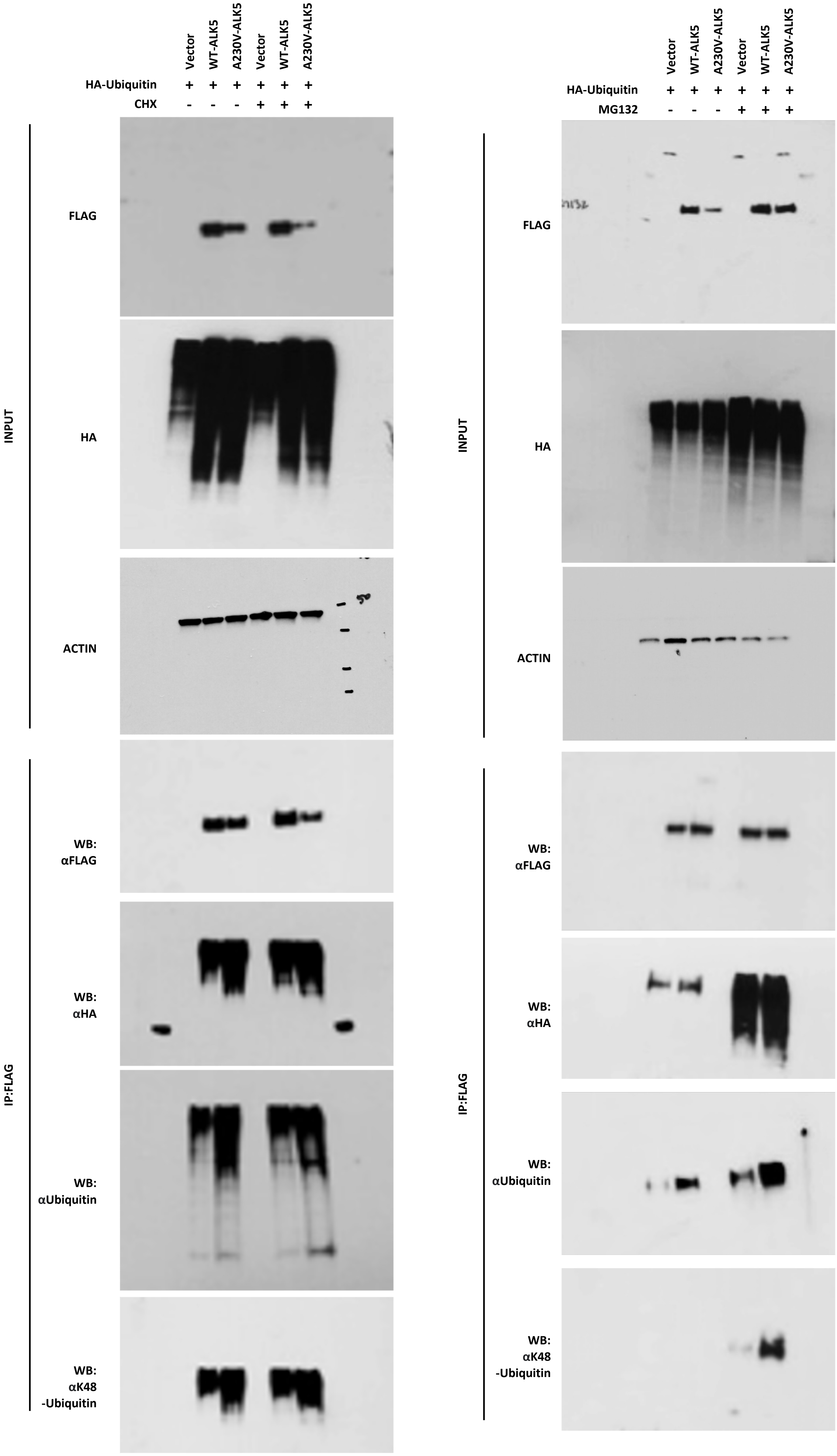

Figure 4A- extended image

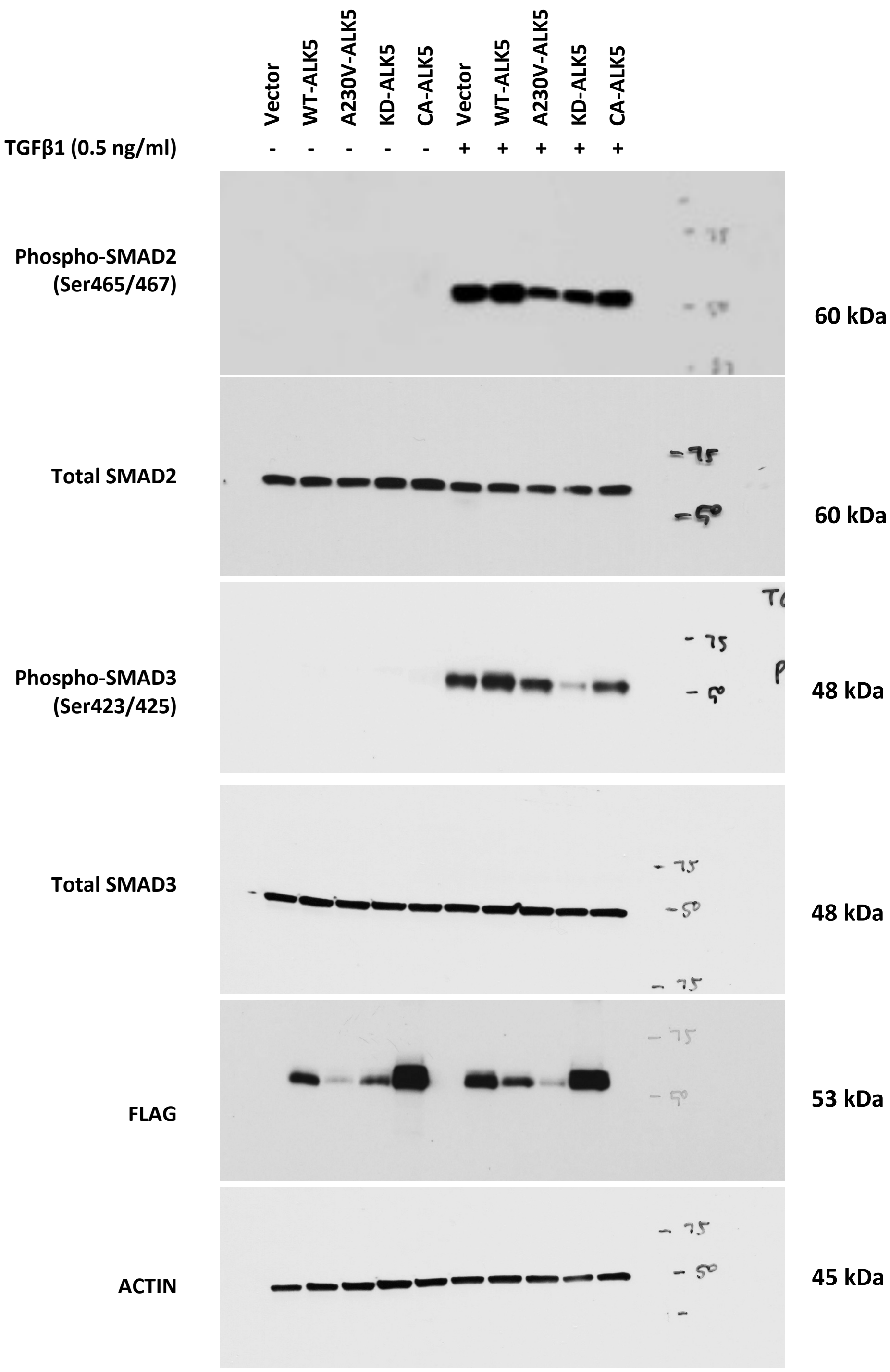

Figure 4B- extended image

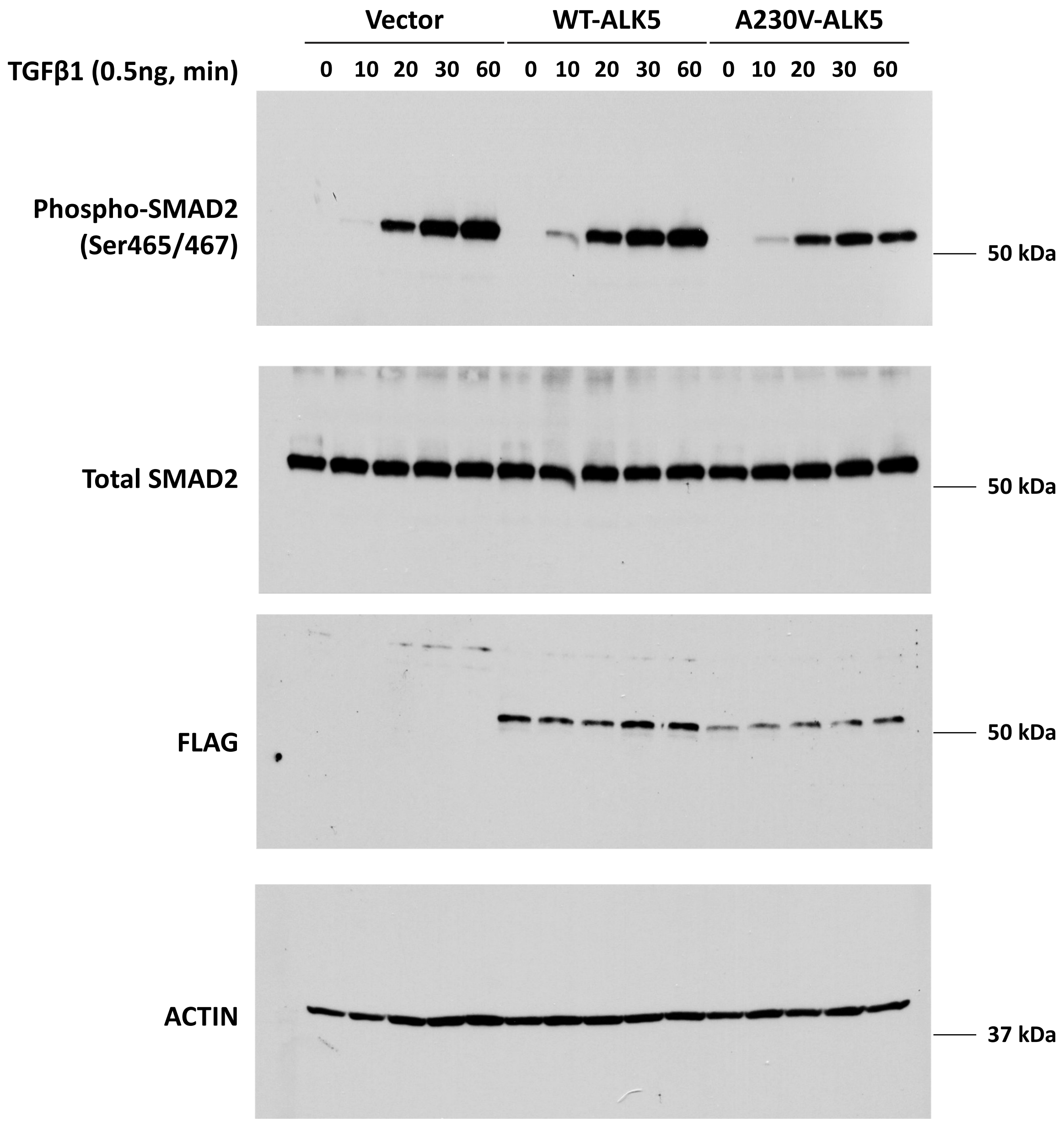

Figure 4C- extended image

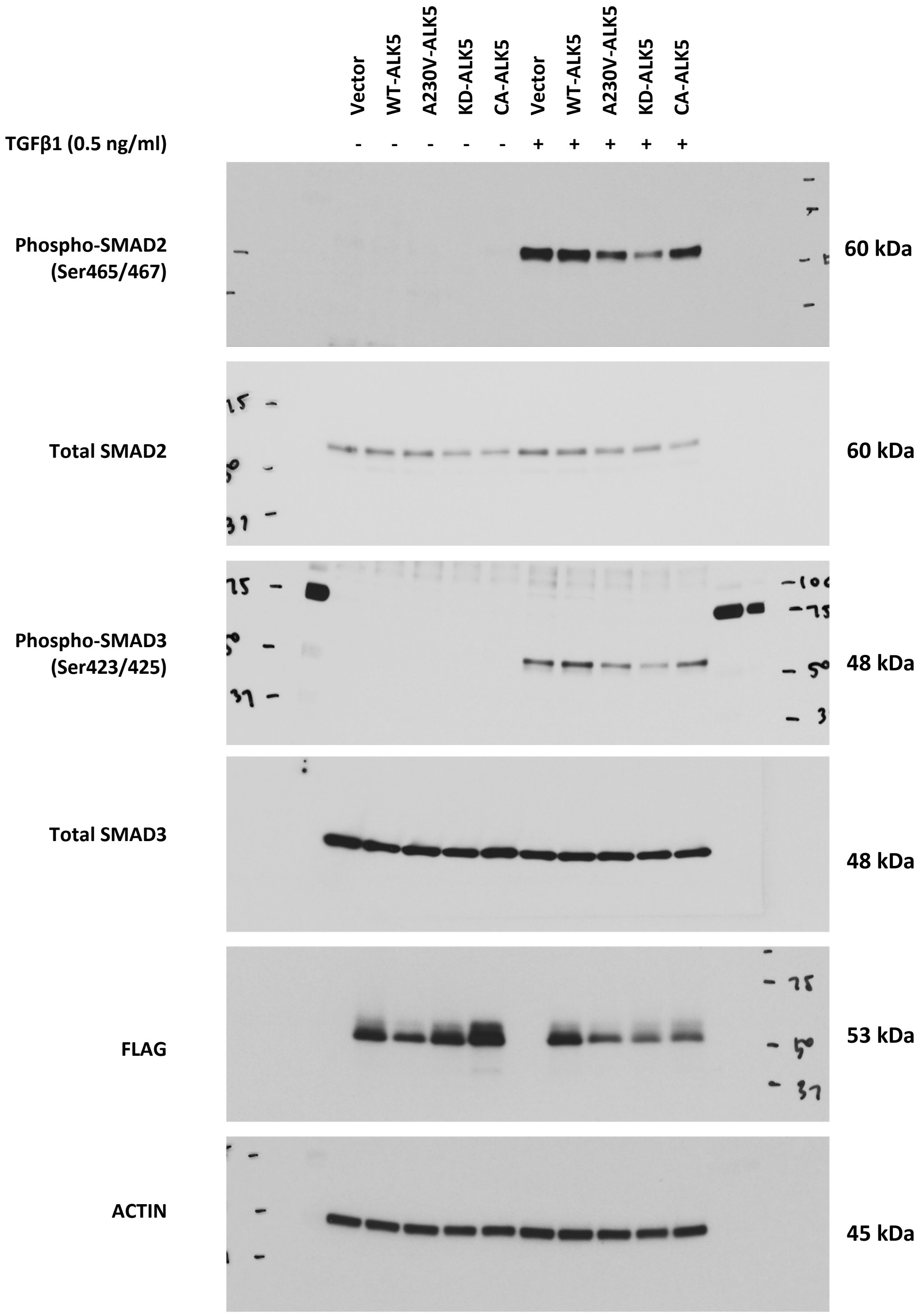

Figure 4D- extended image

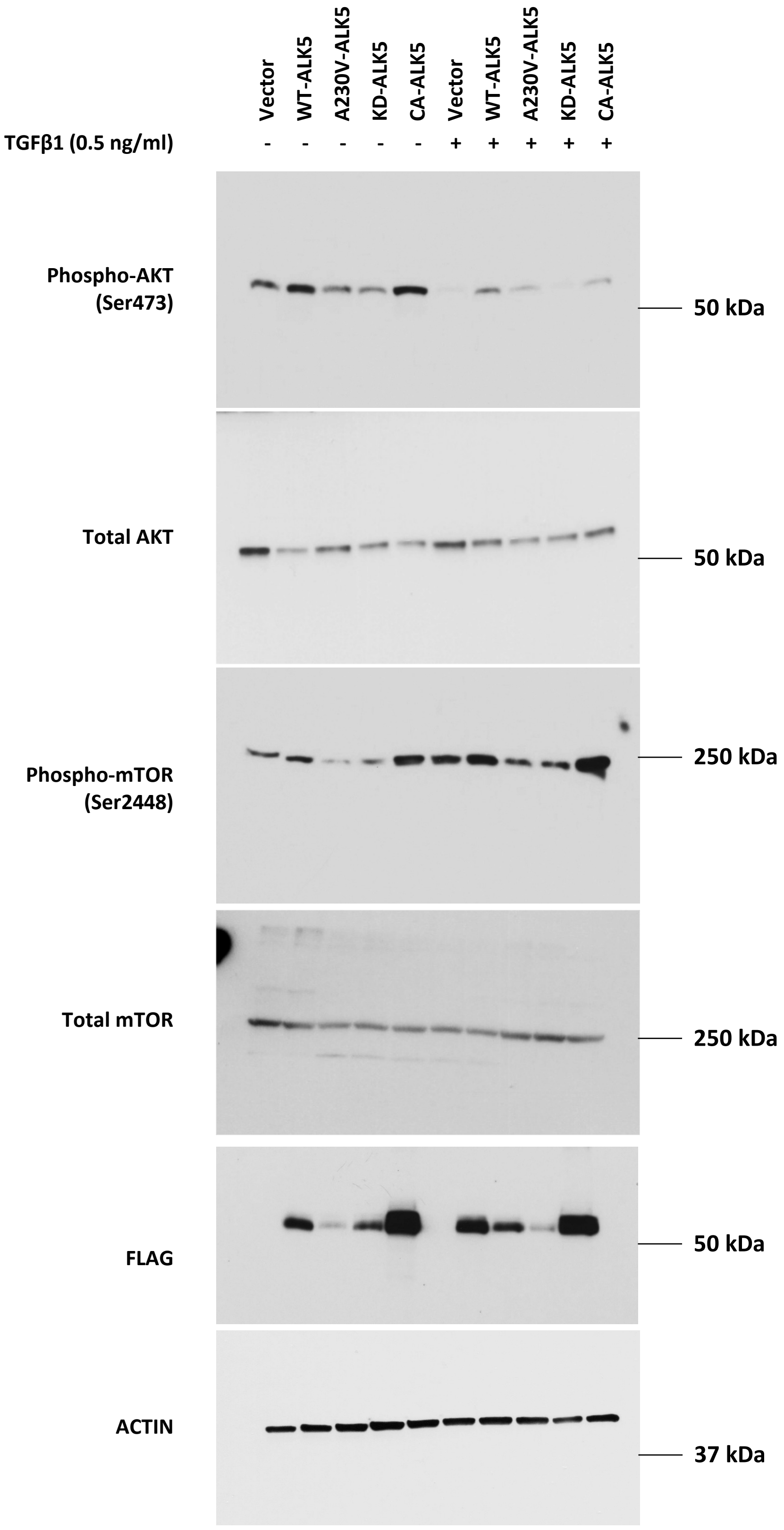

Figure 5C- extended image

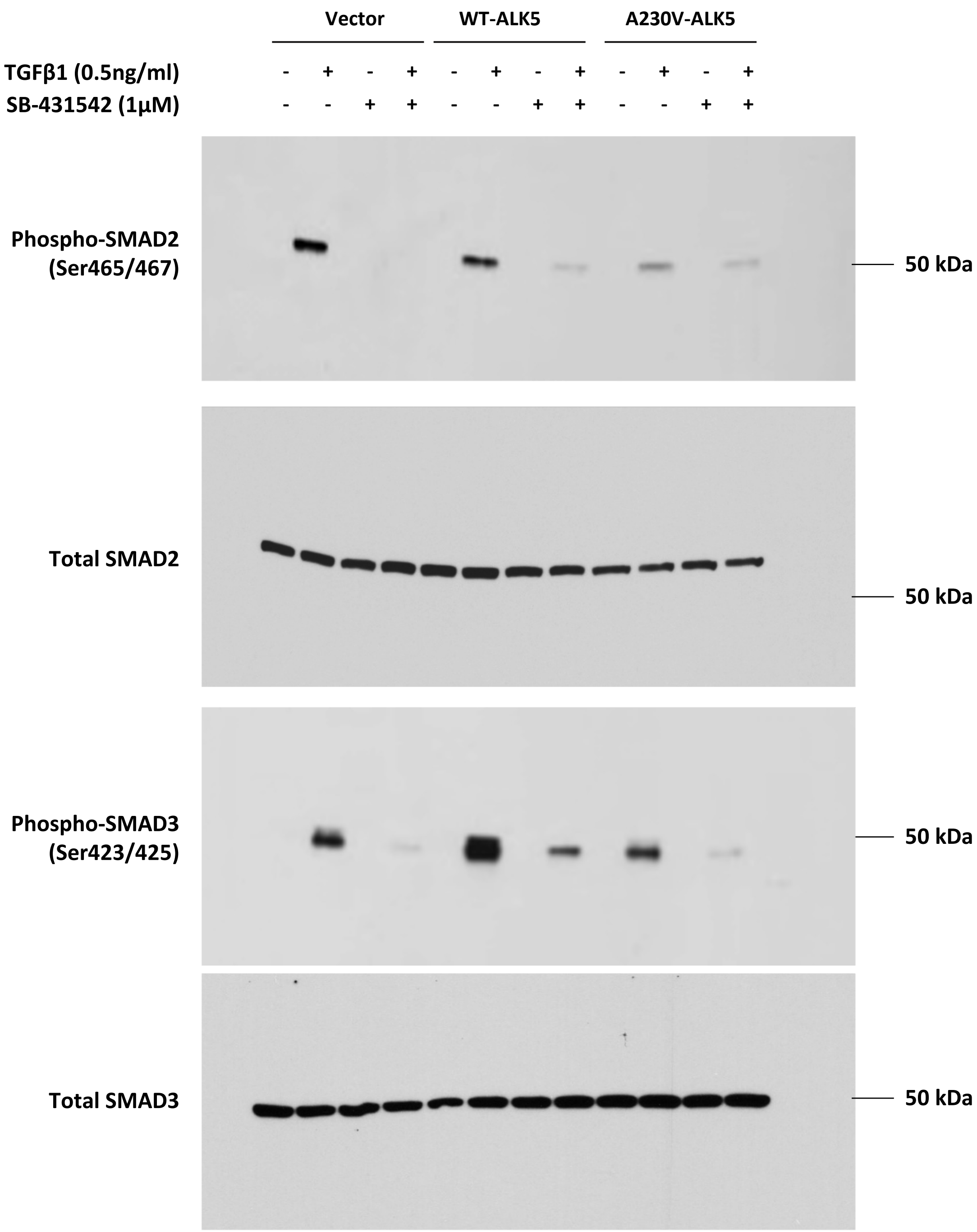

Figure 5C- extended image (continued)

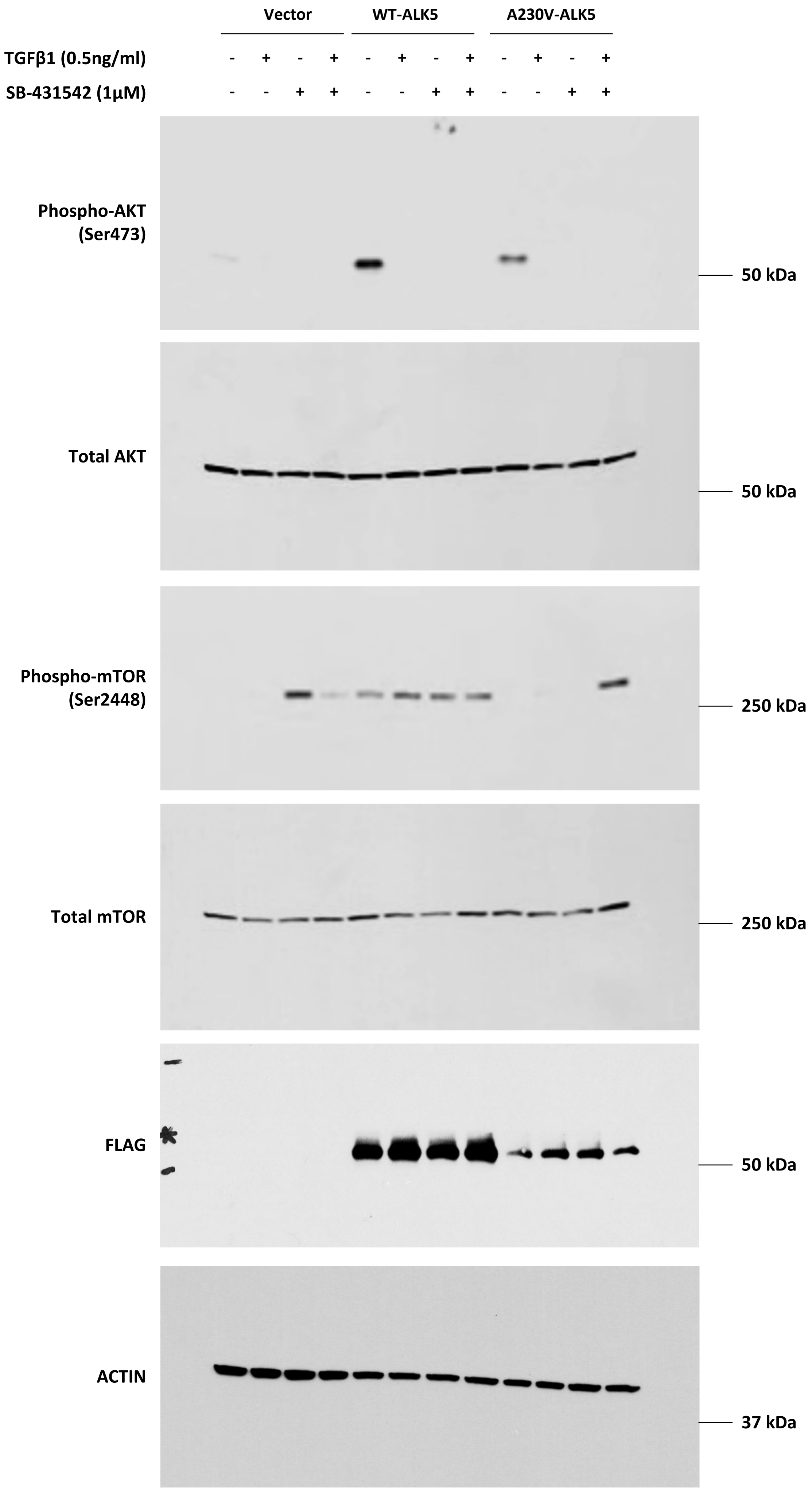

Figure 5D- extended image

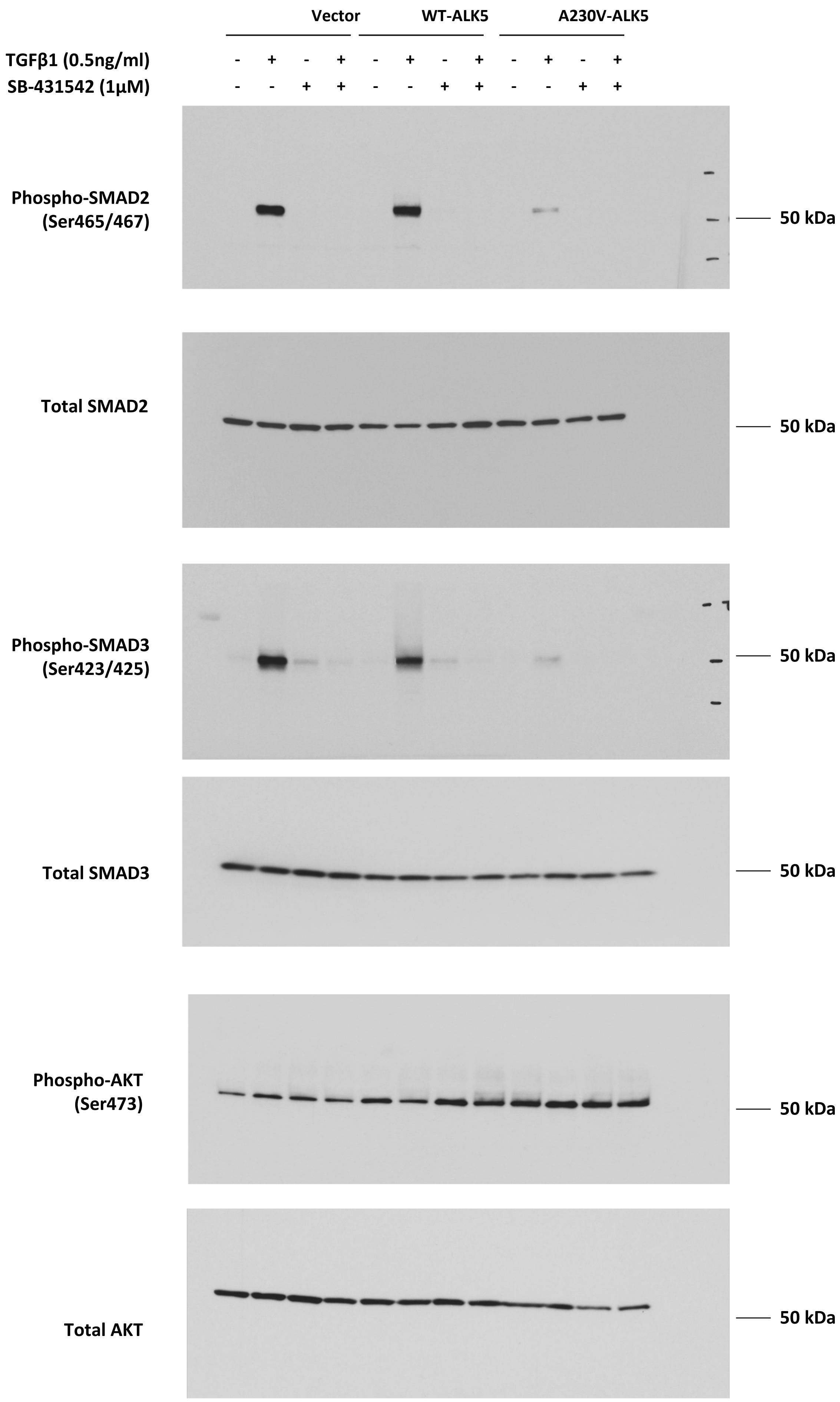

Figure 5C- extended image (continued)

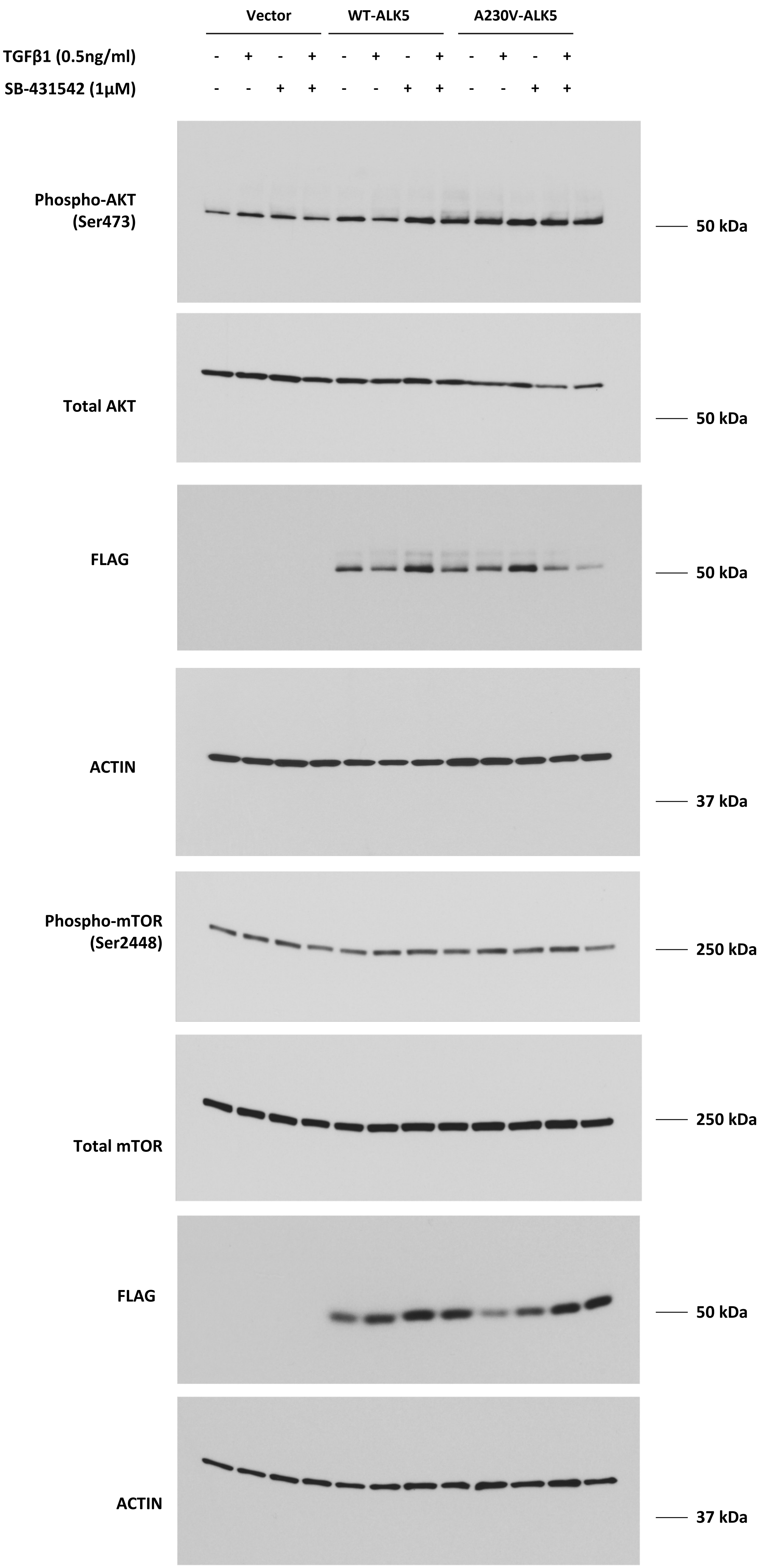

Supplement: S1 Raw images — (PDF) [file pone.0312806.s001.pdf]
