# Supplementary material for: The endometrial cancer A230V-ALK5 (TGFBR1) mutant attenuates TGF-β signaling and exhibits reduced in vitro sensitivity to ALK5 inhibitors
Source: PLoS One. 2024 Nov 22;19(11):e0312806. doi: 10.1371/journal.pone.0312806 (PMC11584080; doi:10.1371/journal.pone.0312806)

Figure 3A- replicate

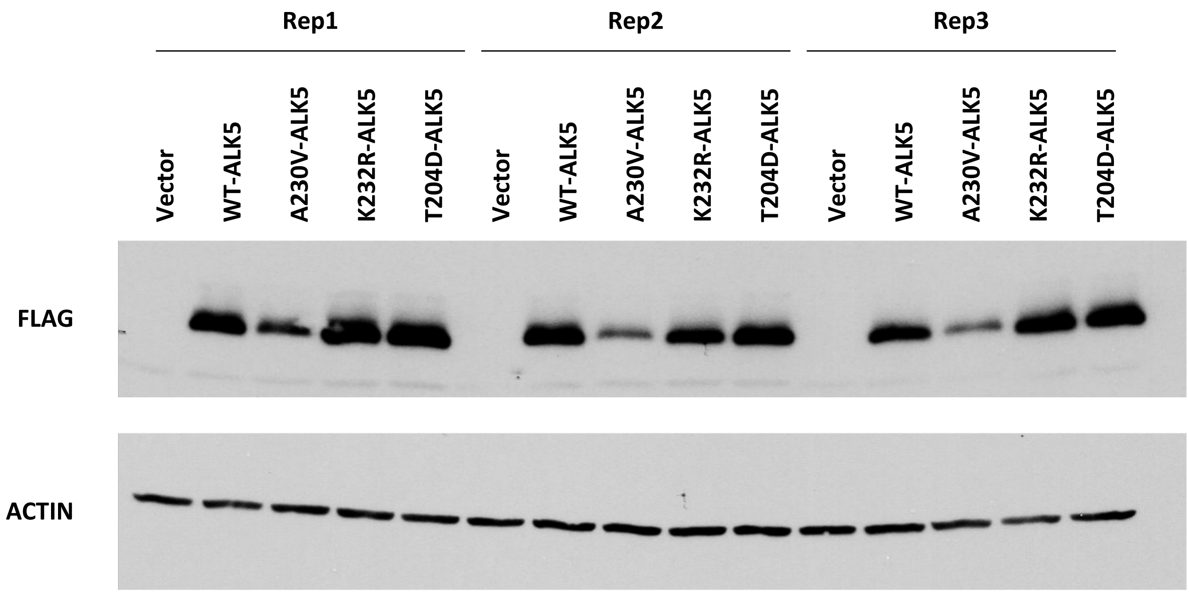

Figure 3B- replicates

Rep 2

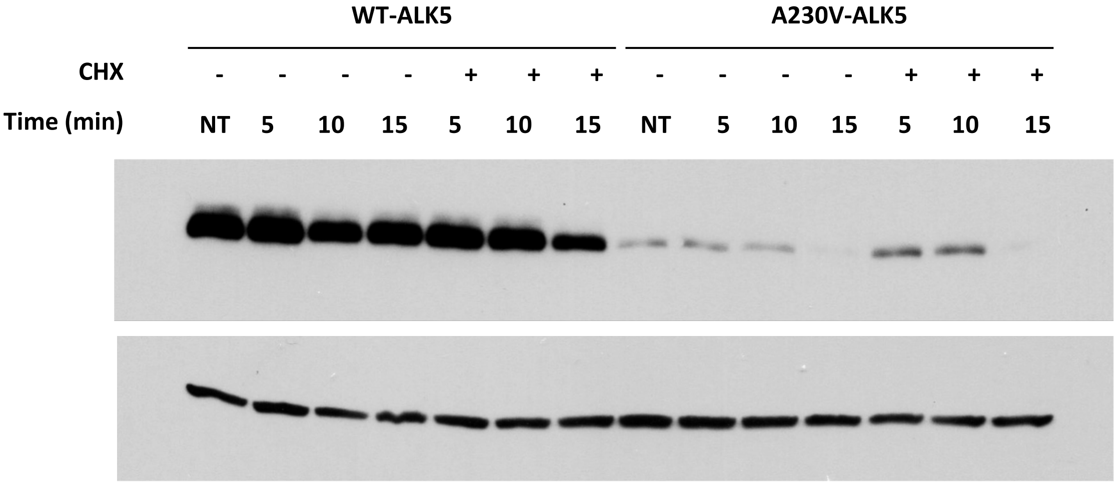

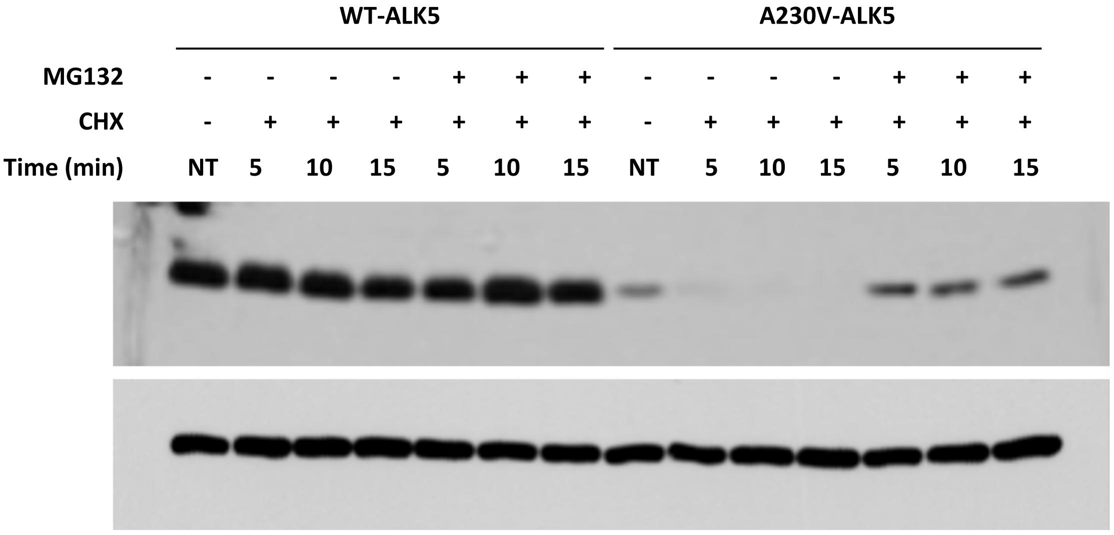

Rep 3

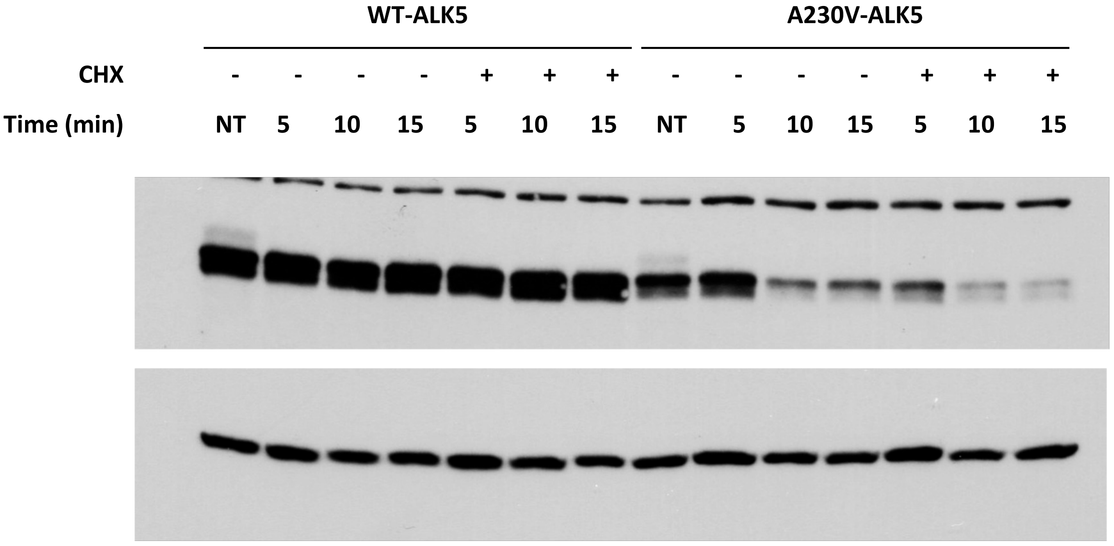

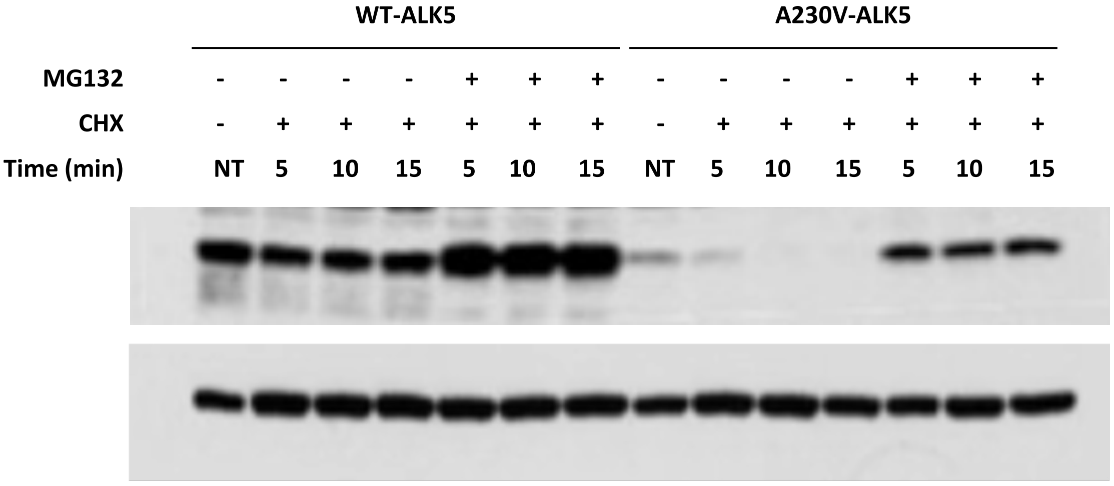

Figure 3C- replicates

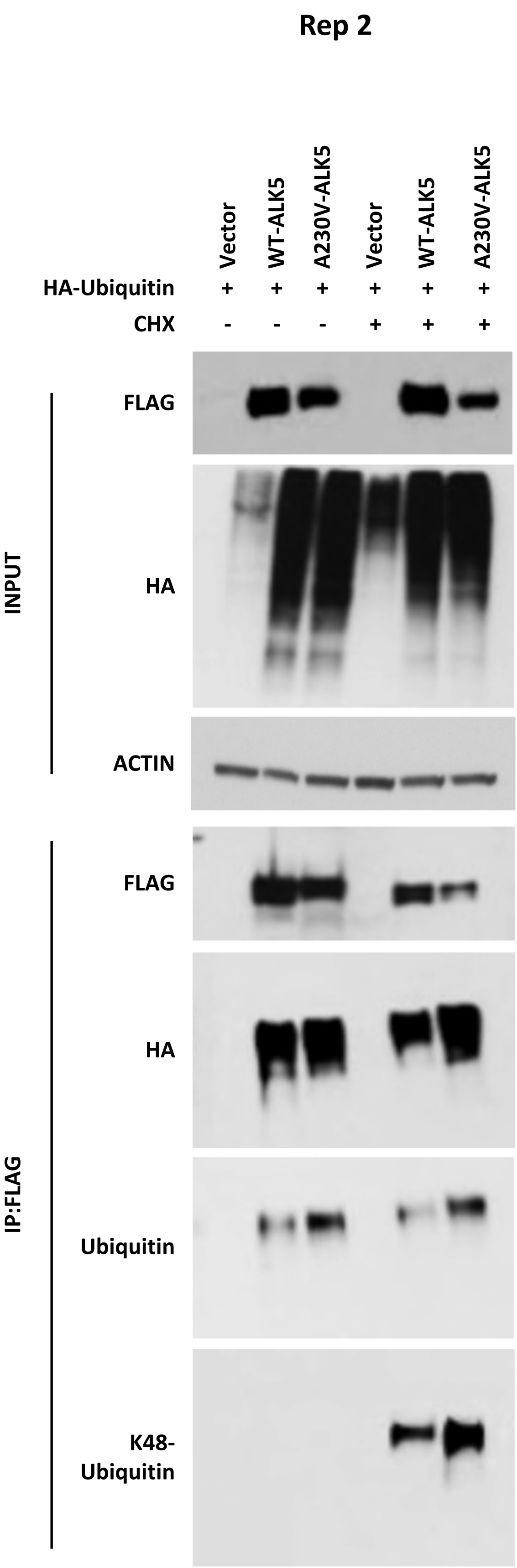

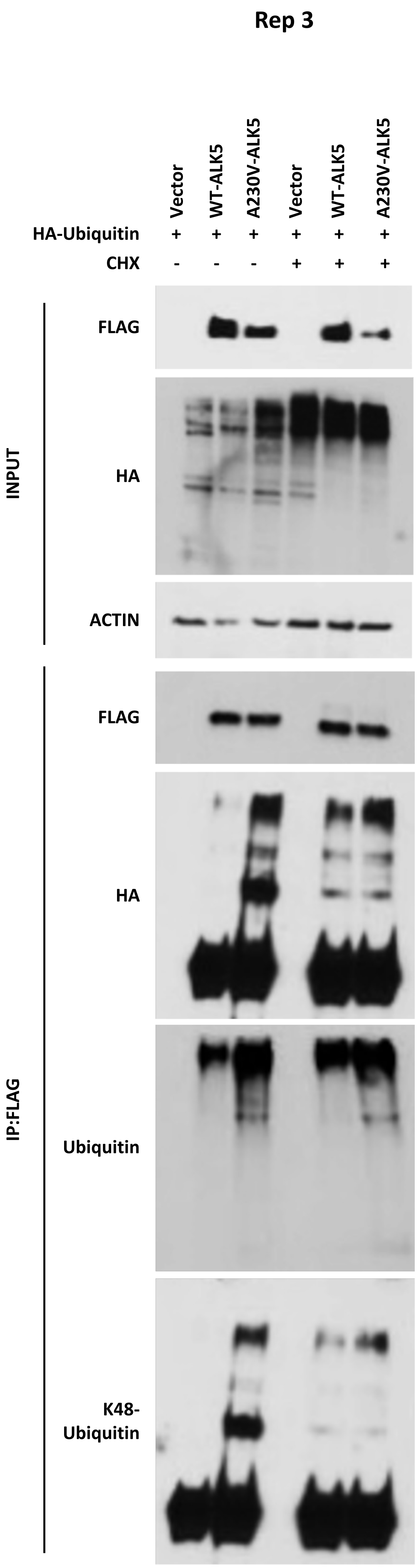

Figure 3D- replicates

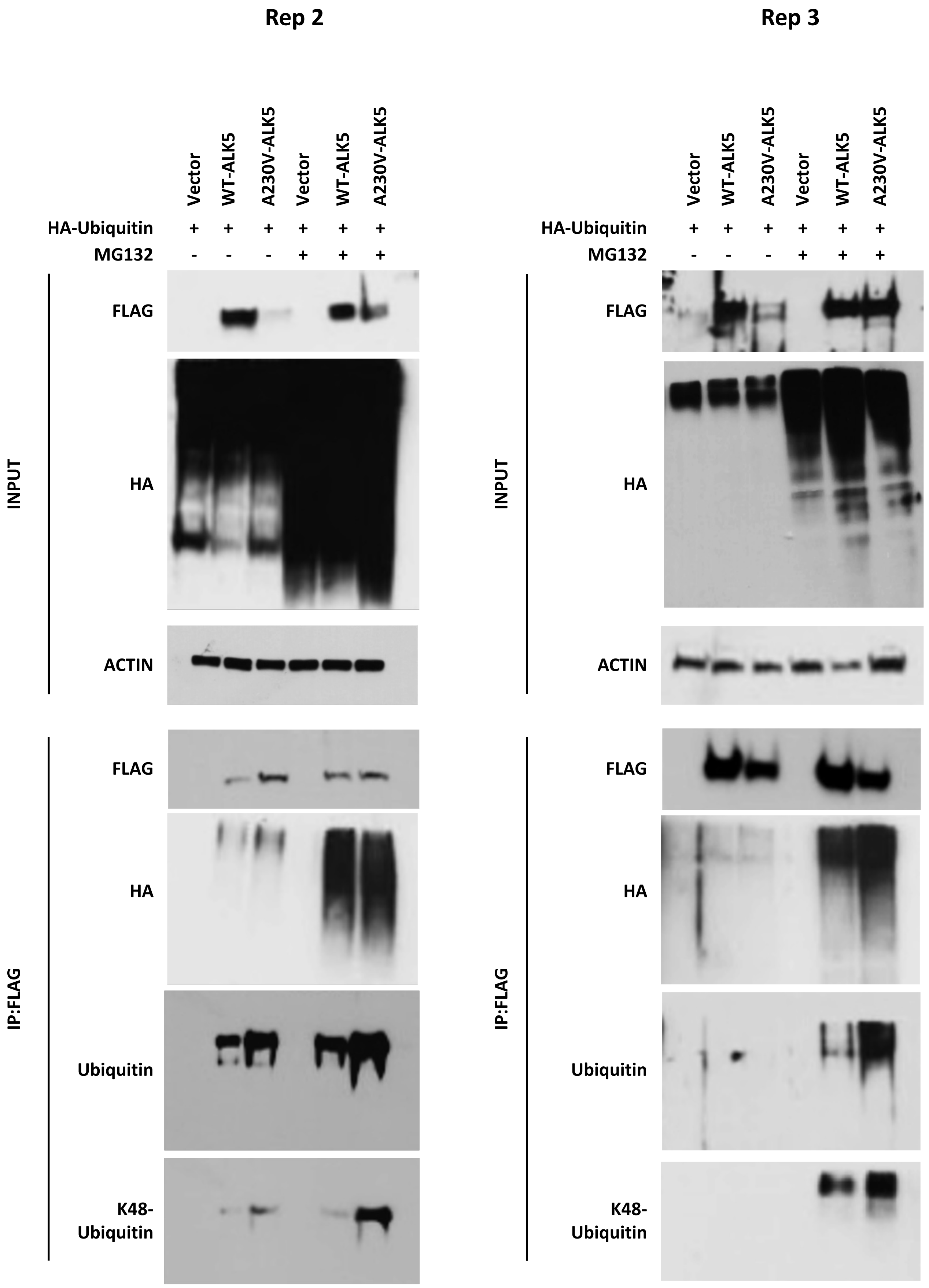

Figure 4A,4D- replicates

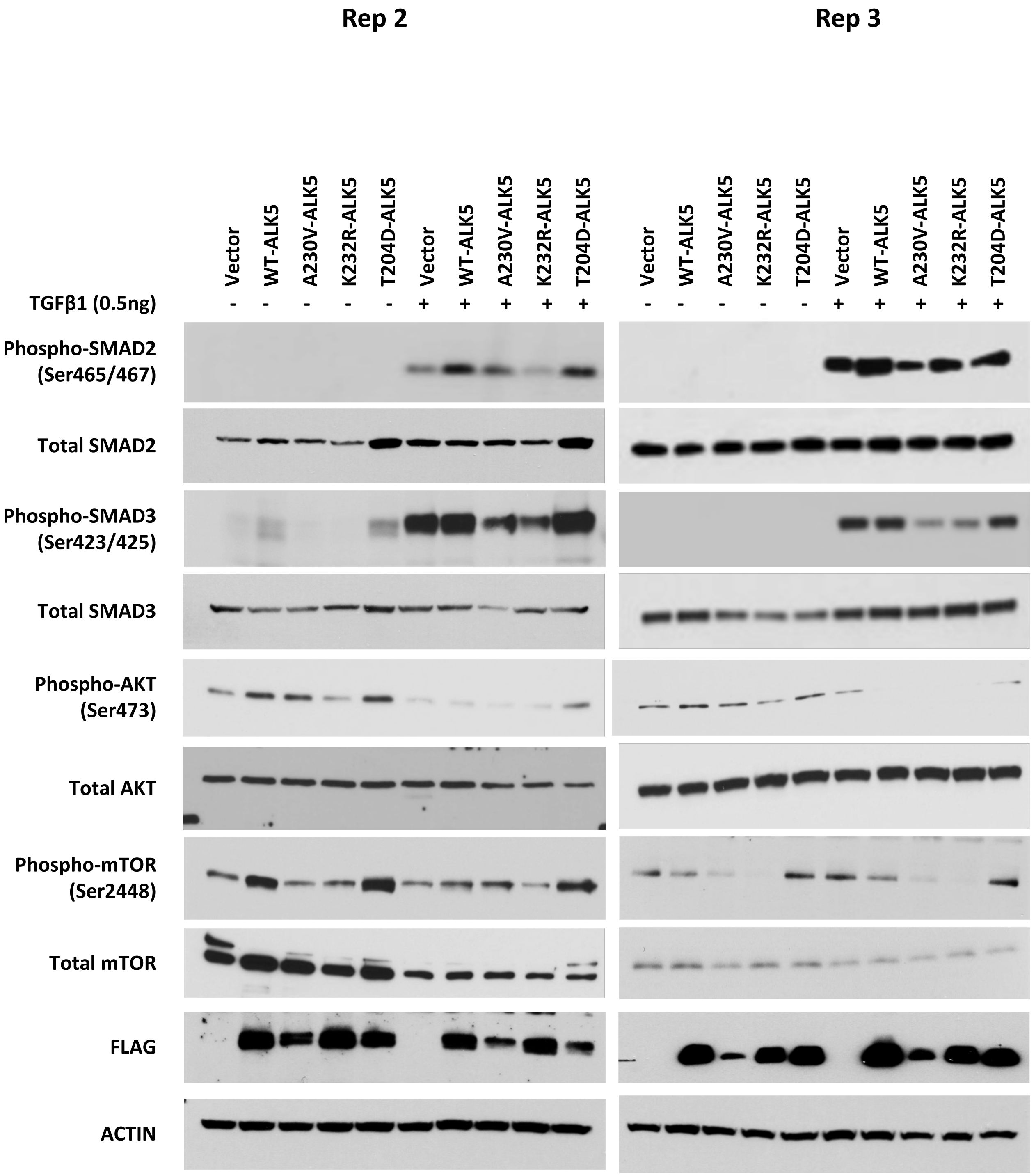

Figure 4B- replicates

Rep 2

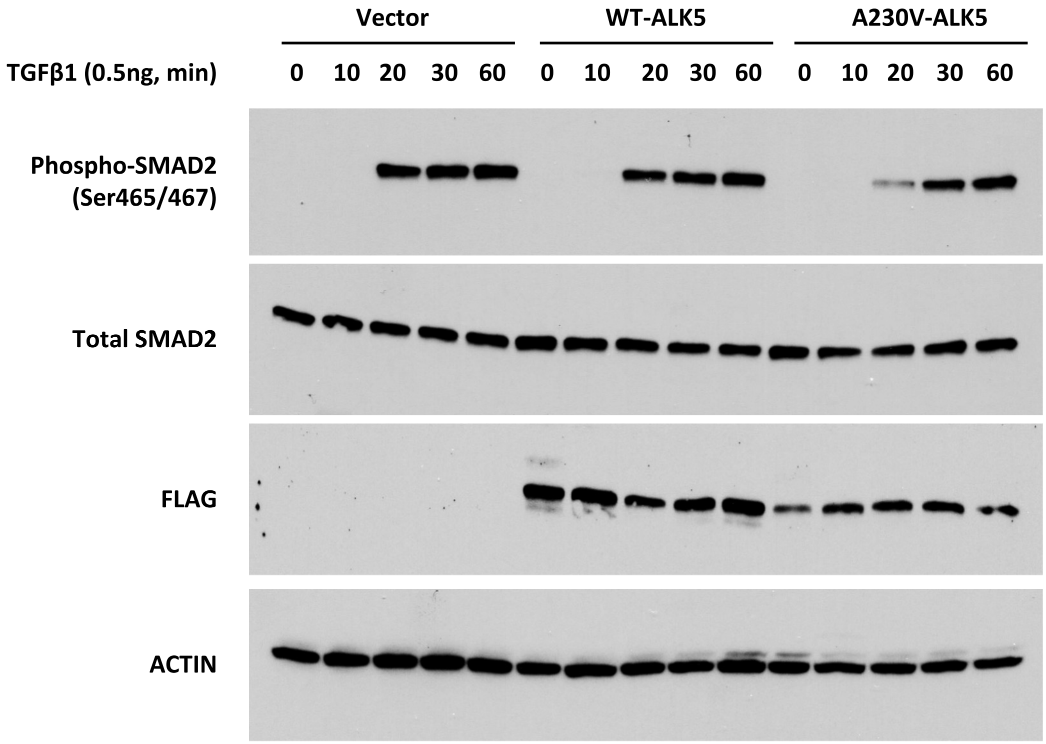

Rep 3

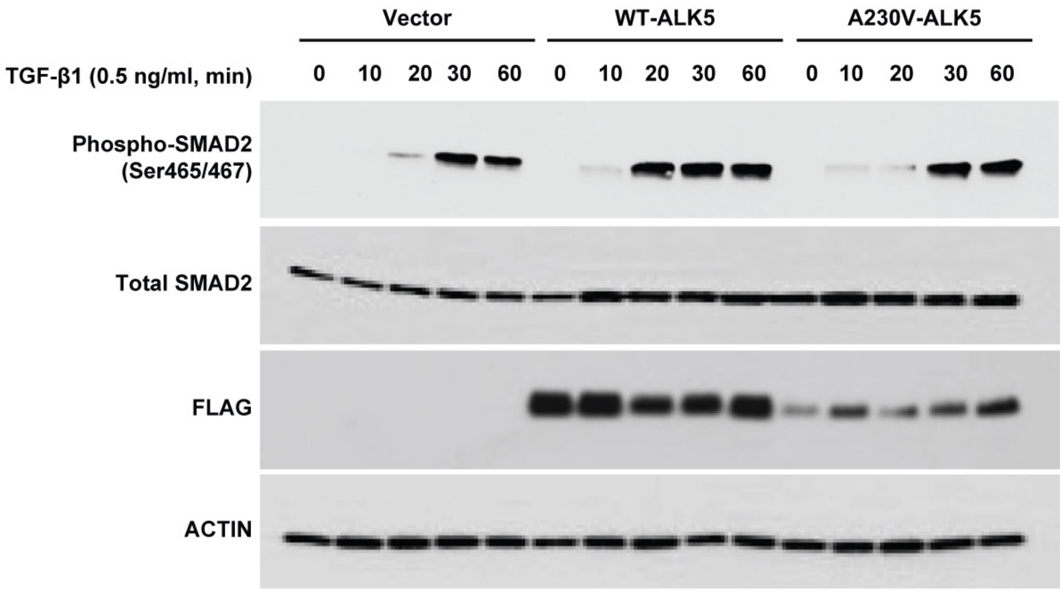

Figure 4C- replicates

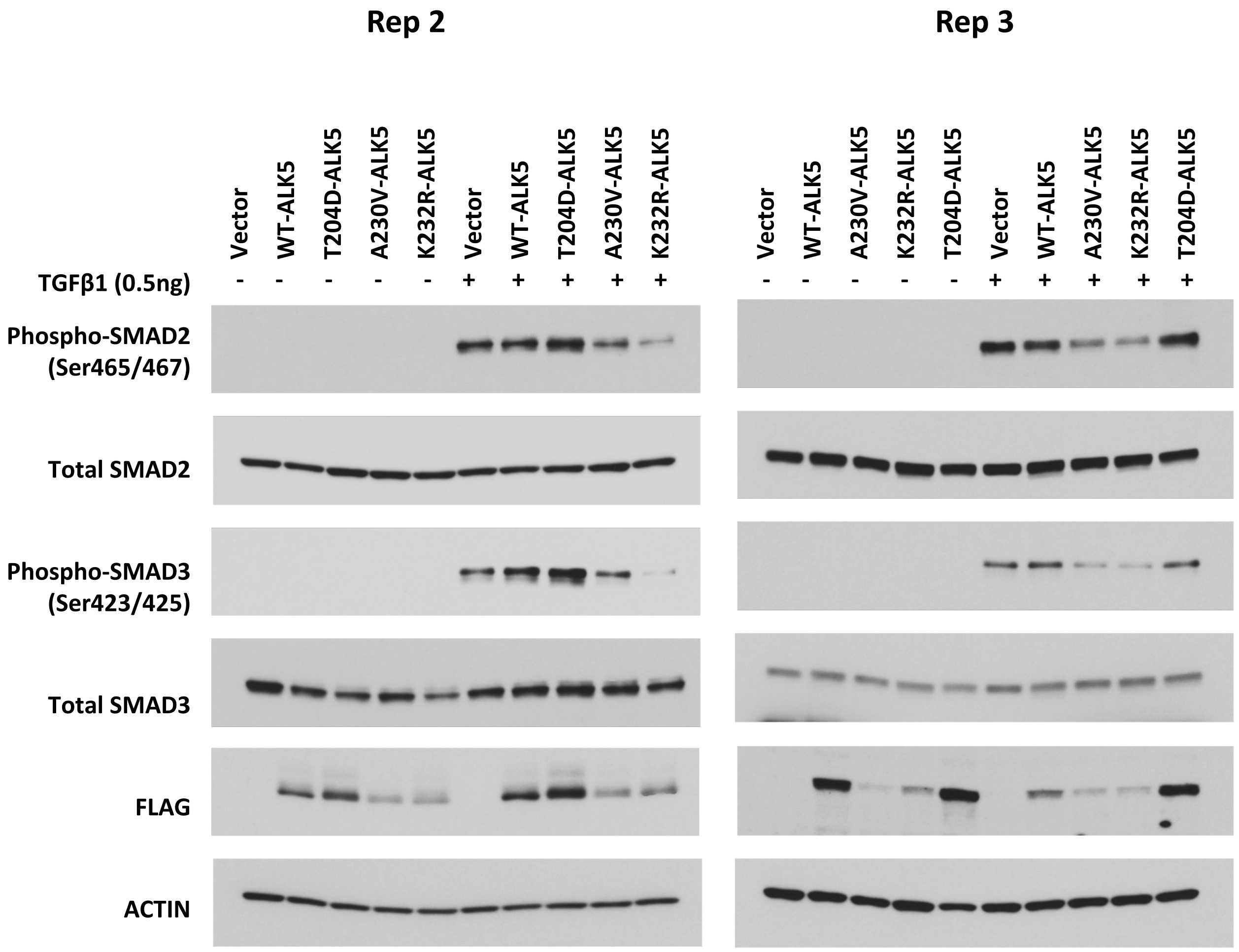

Figure 5C- replicates

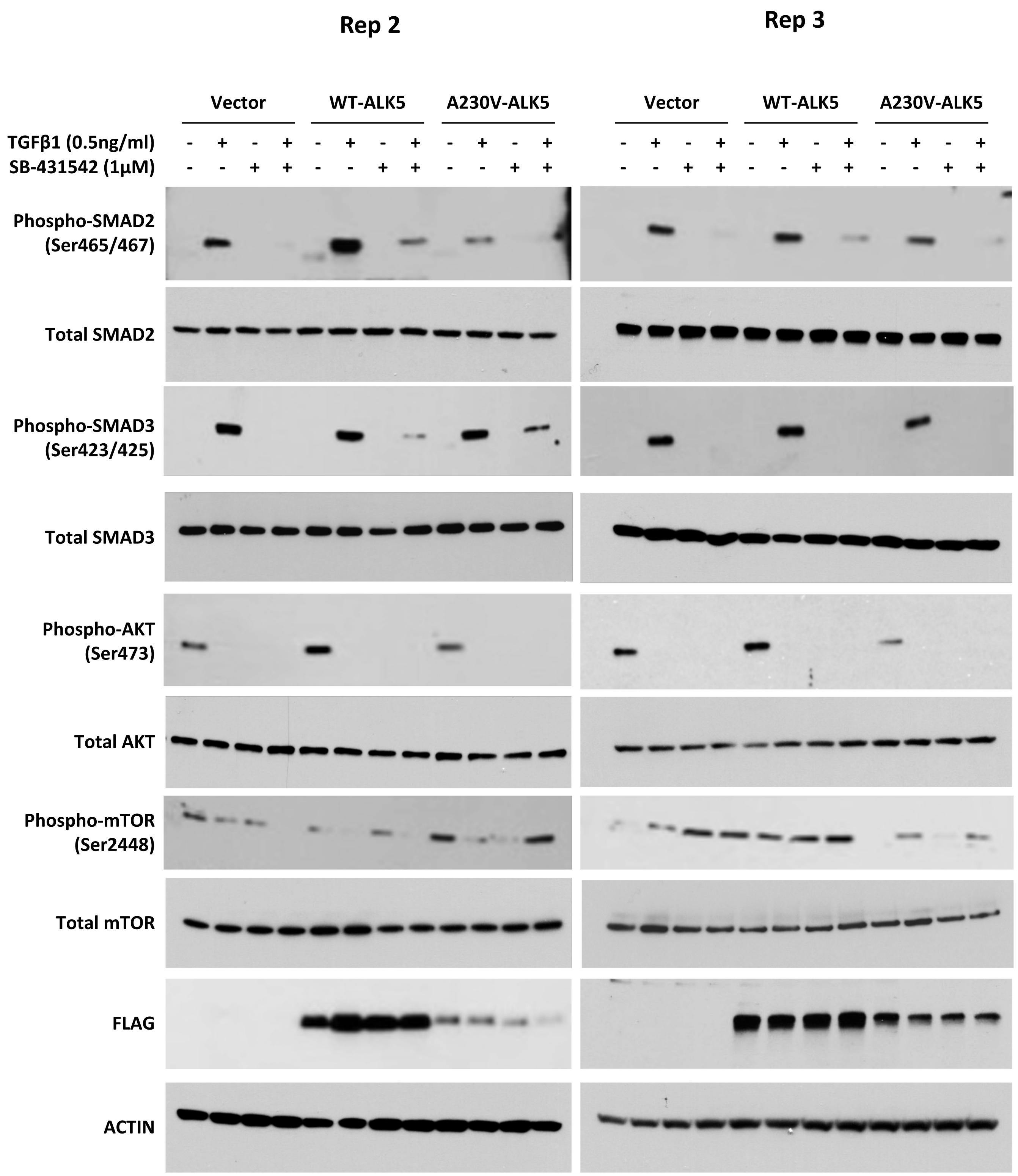

Figure 5D- replicates

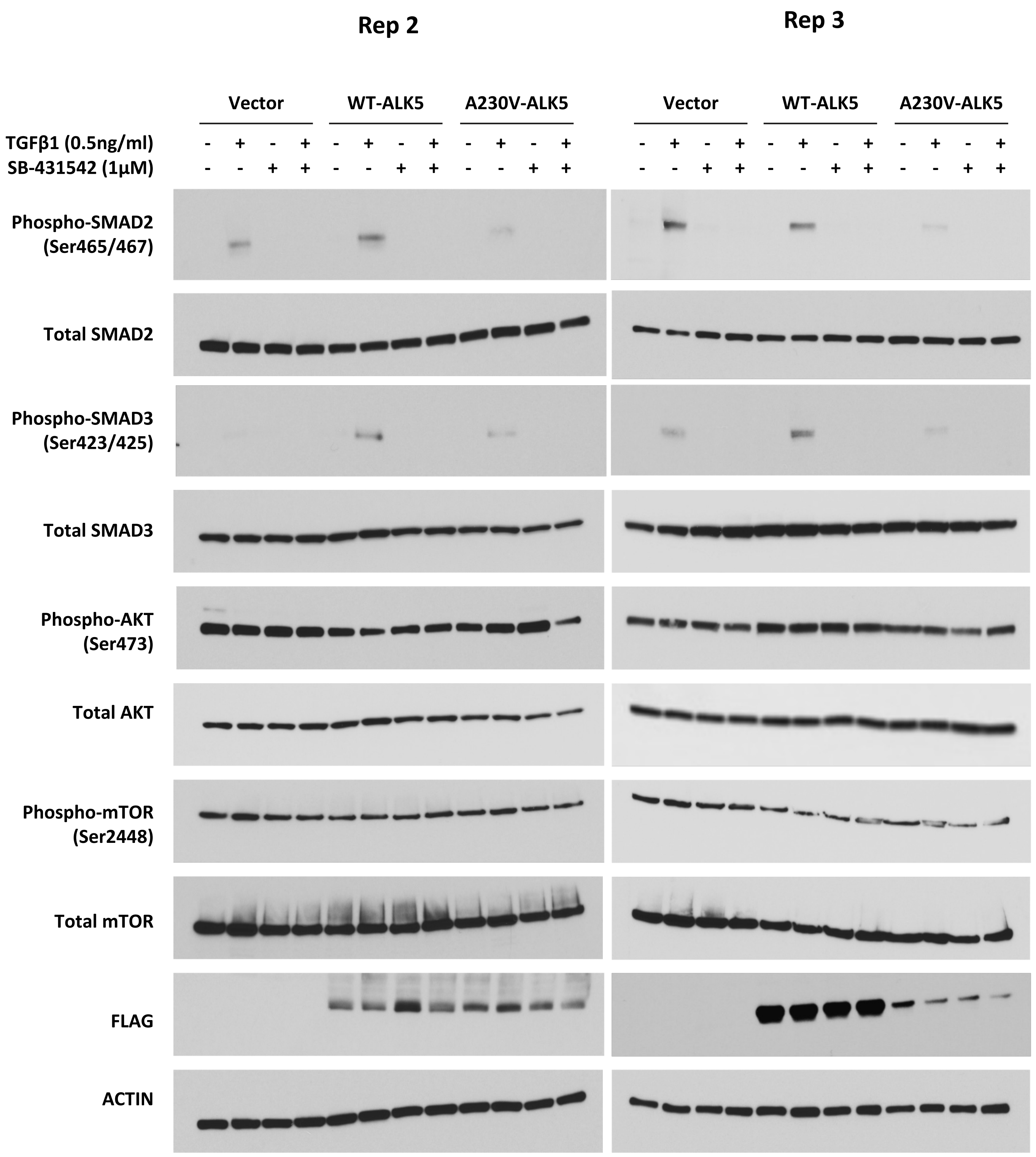

Supplement: S2 Raw images — (PDF) [file pone.0312806.s002.pdf]
