# Supplementary figures and images for: The endometrial cancer A230V-ALK5 (TGFBR1) mutant attenuates TGF-β signaling and exhibits reduced in vitro sensitivity to ALK5 inhibitors
Source: PLoS One. 2024 Nov 22;19(11):e0312806. doi: 10.1371/journal.pone.0312806 (PMC11584080; doi:10.1371/journal.pone.0312806)

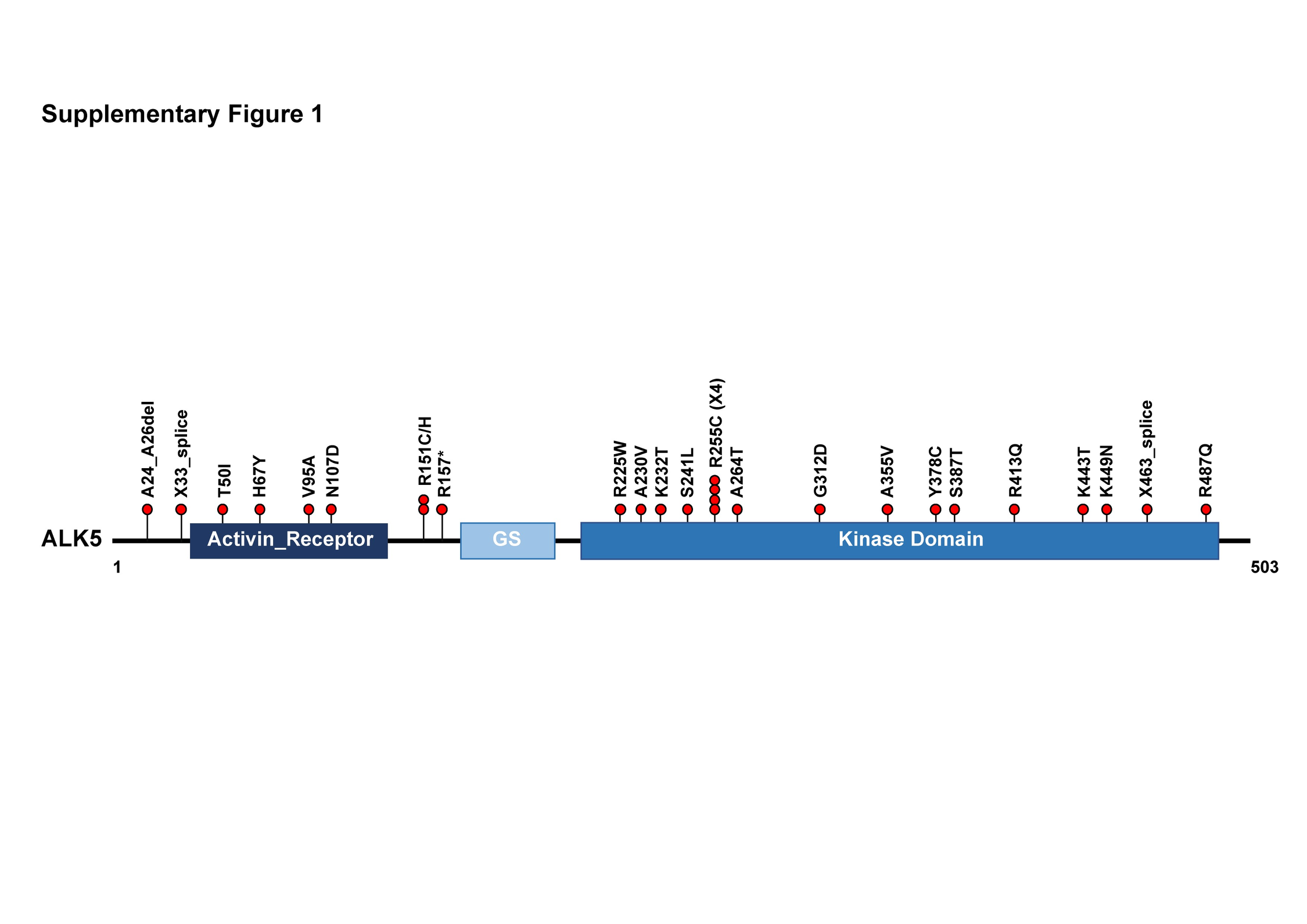

Supplement: S1 Fig — Lollipop plot indicates the positions of somatic mutations relative to ALK5 functional domains. Each circle (red) represents a single mutation; the ALK5-R255C kinase domain mutant is recurrent. Abbreviations: GS, glycine (G) and serine (S) rich sequence. (TIF) [file pone.0312806.s009.tif]

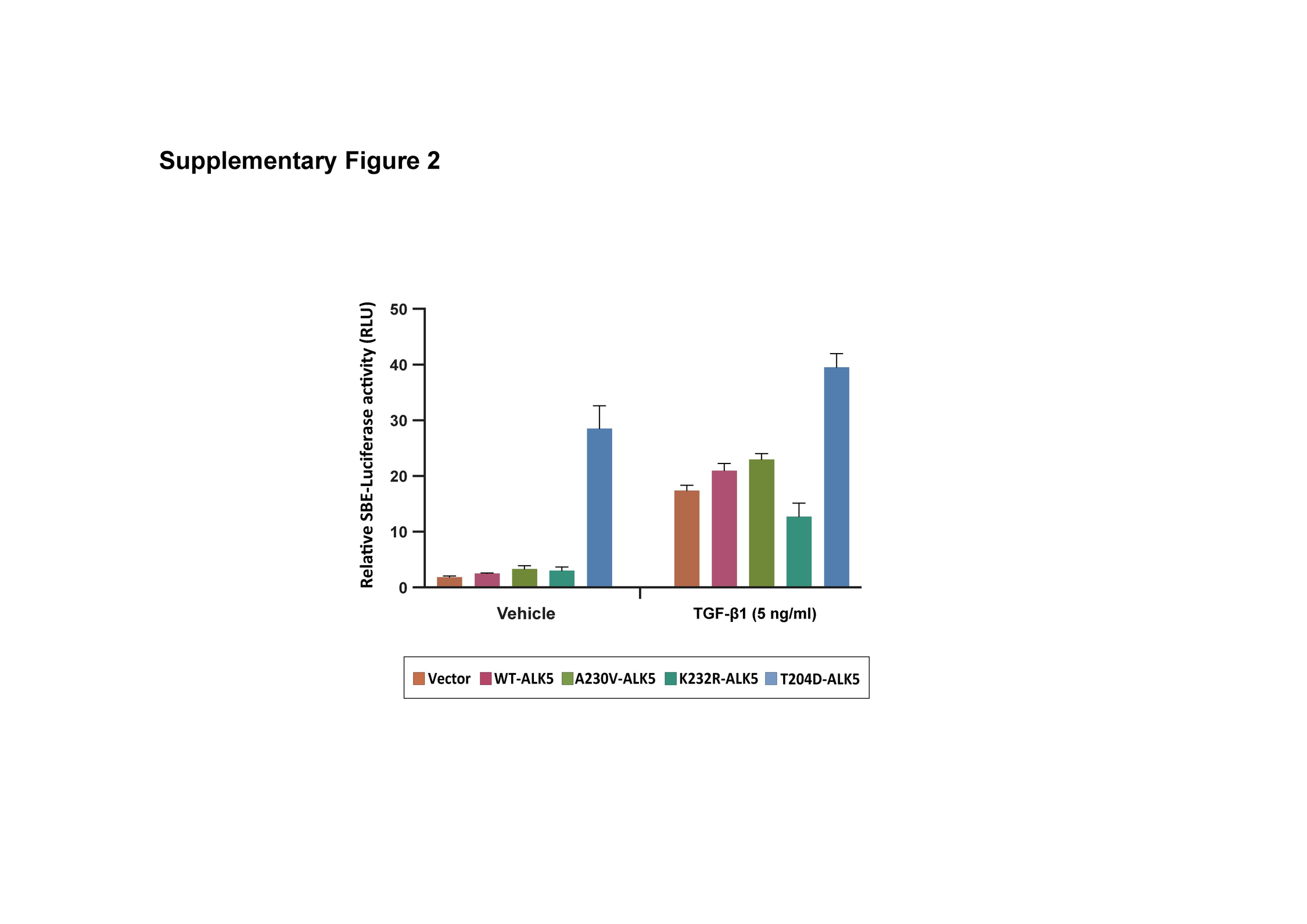

Supplement: S2 Fig — Results of TGF-β1-responsive SMAD binding element (SBE) firefly luciferase reporter assays in NIH/3T3 cells. Cells were triply transfected with pGL4.48[SBE] Firefly luciferase reporter construct, pGL4.74[TK] Renilla vector and either a wildtype (WT) or mutant FLAG-tagged ALK5 expression construct or control vector (CV). Expression constructs containing the ALK5-T204D (constitutively active mutant) or -K232R (kinase-dead) mutant were included as positive and negative controls. After 24 h transfection, cells were serum-starved for 30 min before TGF-β1 was added at the indicated concentration for an additional 3 h and luciferase activity was measured after the addition of assay reagents based on manufacturer’s protocol (Dual-Glo luciferase assay system). Relative Luciferase Units were calculated by normalizing SBE luciferase activity to Renilla luciferase activity. The mean ± SD values, based on results of four independent experiments, were calculated using Prism software (GraphPad, Boston, MA) and plotted. P-values were calculated using Two-Way ANOVA; *P<0.05. (TIF) [file pone.0312806.s010.tif]

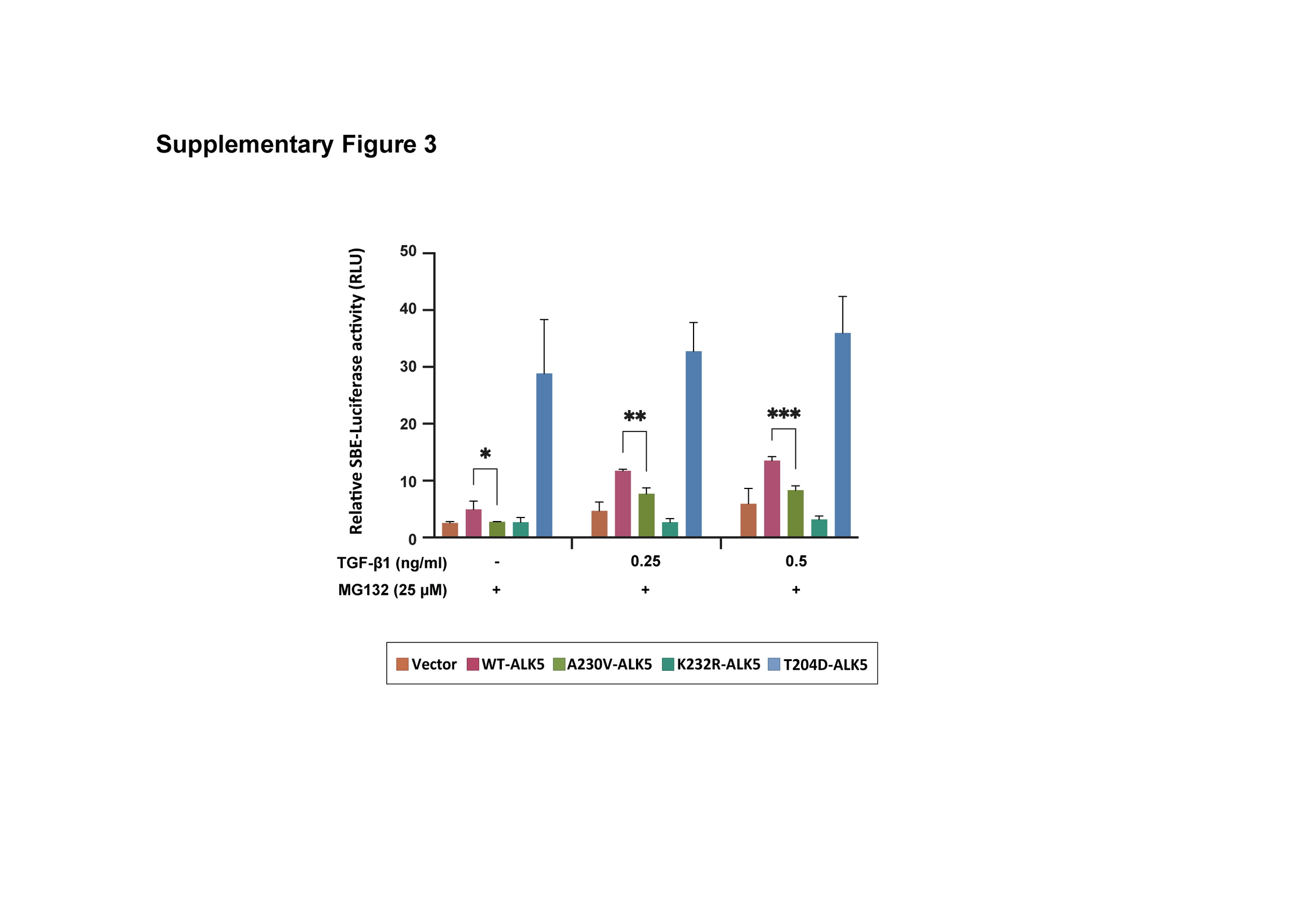

Supplement: S3 Fig — Results of TGF-β1-responsive SMAD binding element (SBE) firefly luciferase reporter assays in NIH/3T3 cells, following MG132 treatment. Cells were triply transfected with pGL4.48[SBE] Firefly luciferase reporter construct, pGL4.74[TK] Renilla vector and either a wildtype (WT) or mutant FLAG-tagged ALK5 expression construct or control vector (CV). Expression constructs containing the ALK5-T204D (constitutively-active mutant) or -K232R (kinase-dead) mutants were included as positive and negative controls. After 24 h transfection, cells were serum-starved for 30 min before treatment with MG132 for 1 h, followed by TGF-β1 treatment for 3 h. RLU was measured using Dual-Glo luciferase assay system and calculated by normalizing SBE luciferase activity to Renilla luciferase activity. The mean ± SD, based on results of four independent experiments were calculated using Prism software (GraphPad, Boston, MA) and plotted. P-values were calculated using Two-Way ANOVA; *P<0.05. (TIF) [file pone.0312806.s011.tif]

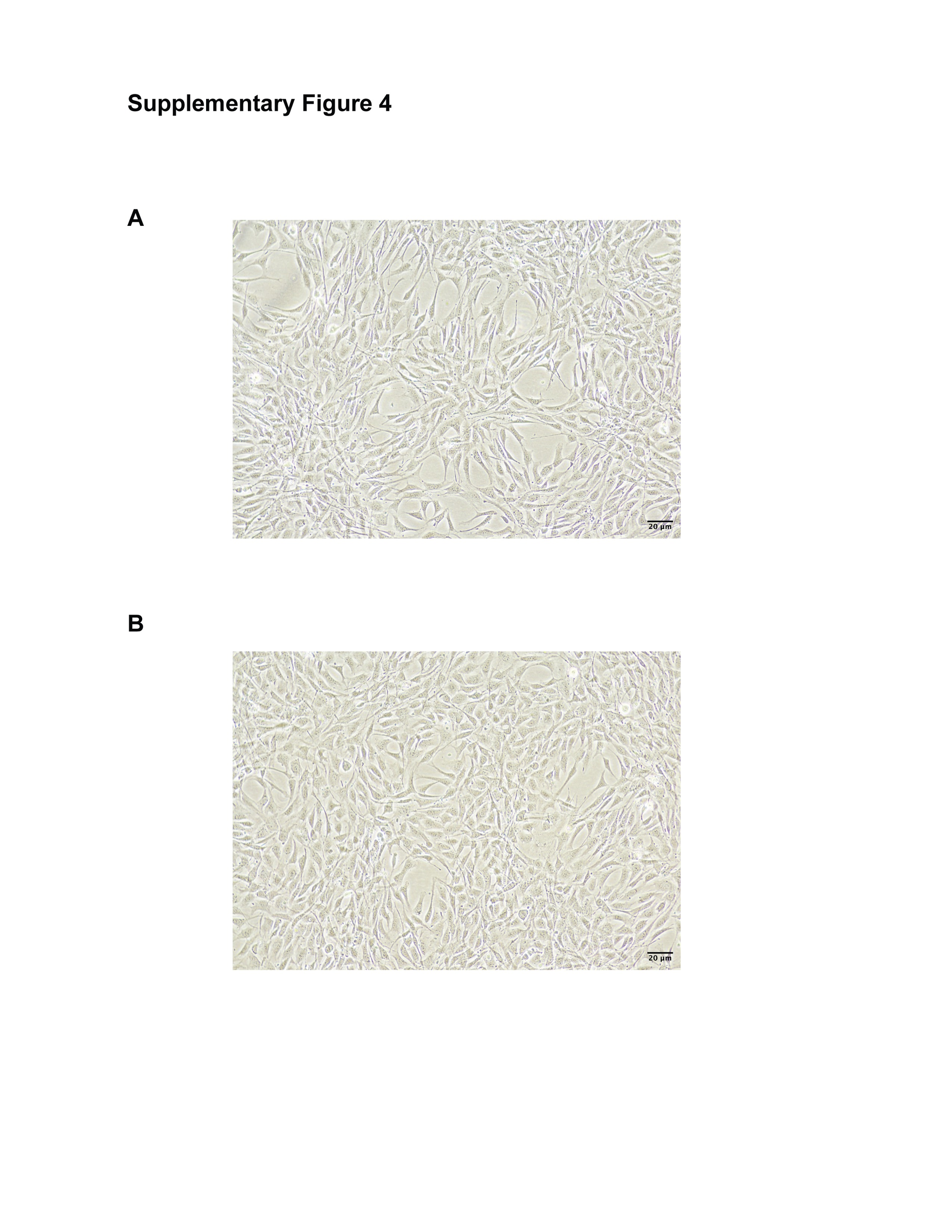

Supplement: S4 Fig — NIH/3T3 cells were transiently transfected with FLAG-tagged ALK5 expression constructs. There were no visible differences in cell morphology 24 h after transfection between (A) WT and (B) A230V mutant cells (10X magnification). (TIF) [file pone.0312806.s012.tif]

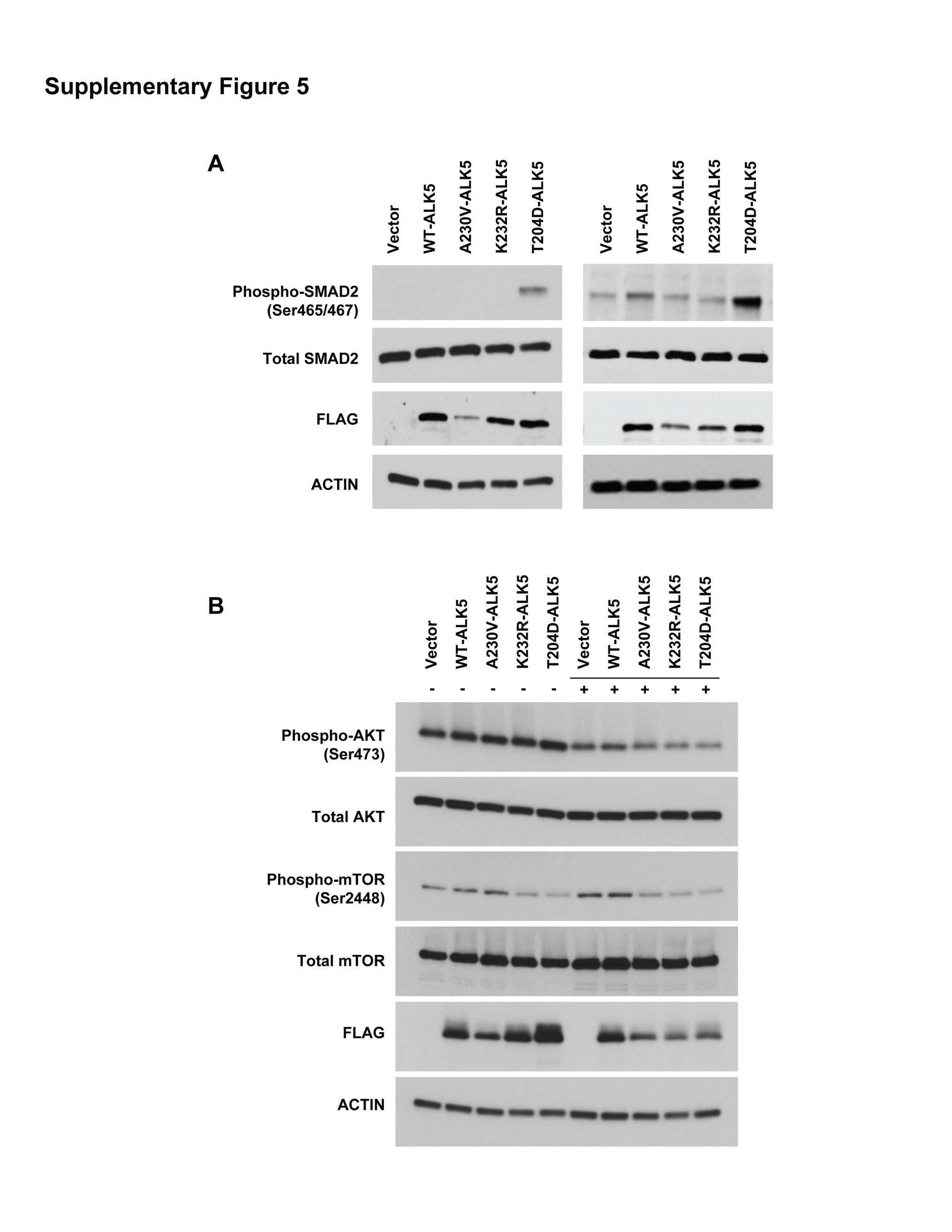

Supplement: S5 Fig — (A) Immunoblots measuring levels of phosphorylated-SMAD2 in NIH/3T3 cells transiently transfected with control vector or the indicated FLAG-ALK5 expression constructs, in serum (2%)-deprived culture medium (left) or complete (10%) serum (right). (B) Immunoblots measuring levels of phosphorylated-AKT and phosphorylated-mTOR in HEEC-265 cells transiently transfected with control vector or the indicated FLAG-ALK5 expression constructs. (TIF) [file pone.0312806.s013.tif]

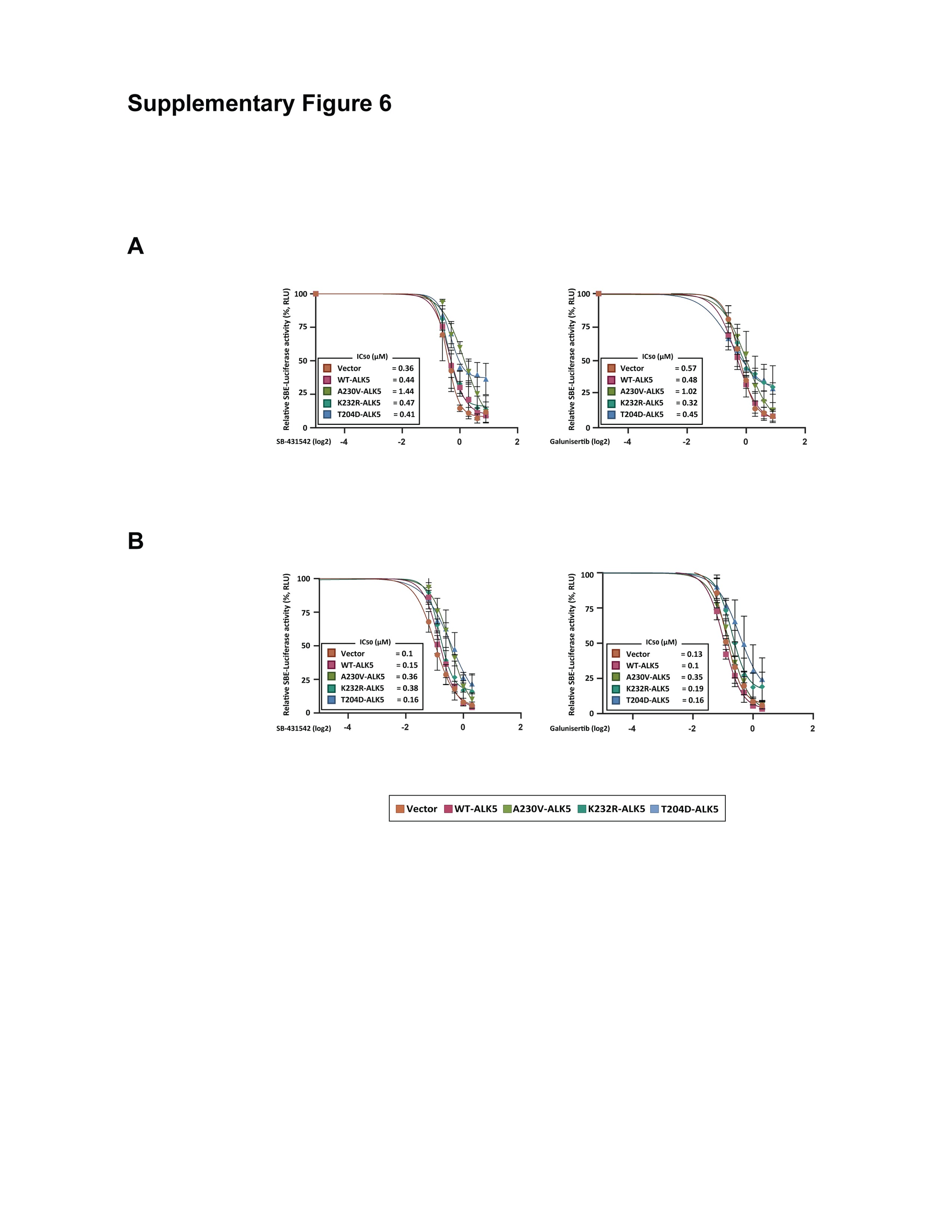

Supplement: S6 Fig — IC50 values for the ALK5 inhibitors SB-431542 and Galunisertib based on luciferase reporter assays conducted in (A) NIH/3T3 cells and (B) HEC-265 cells transfected with the indicated ALK5 constructs and treated with TGF-β1. Nonlinear regression curve fitting was performed using Prism software to plot each graph from four independent experiments. RLU: relative luciferase units. (TIF) [file pone.0312806.s014.tif]

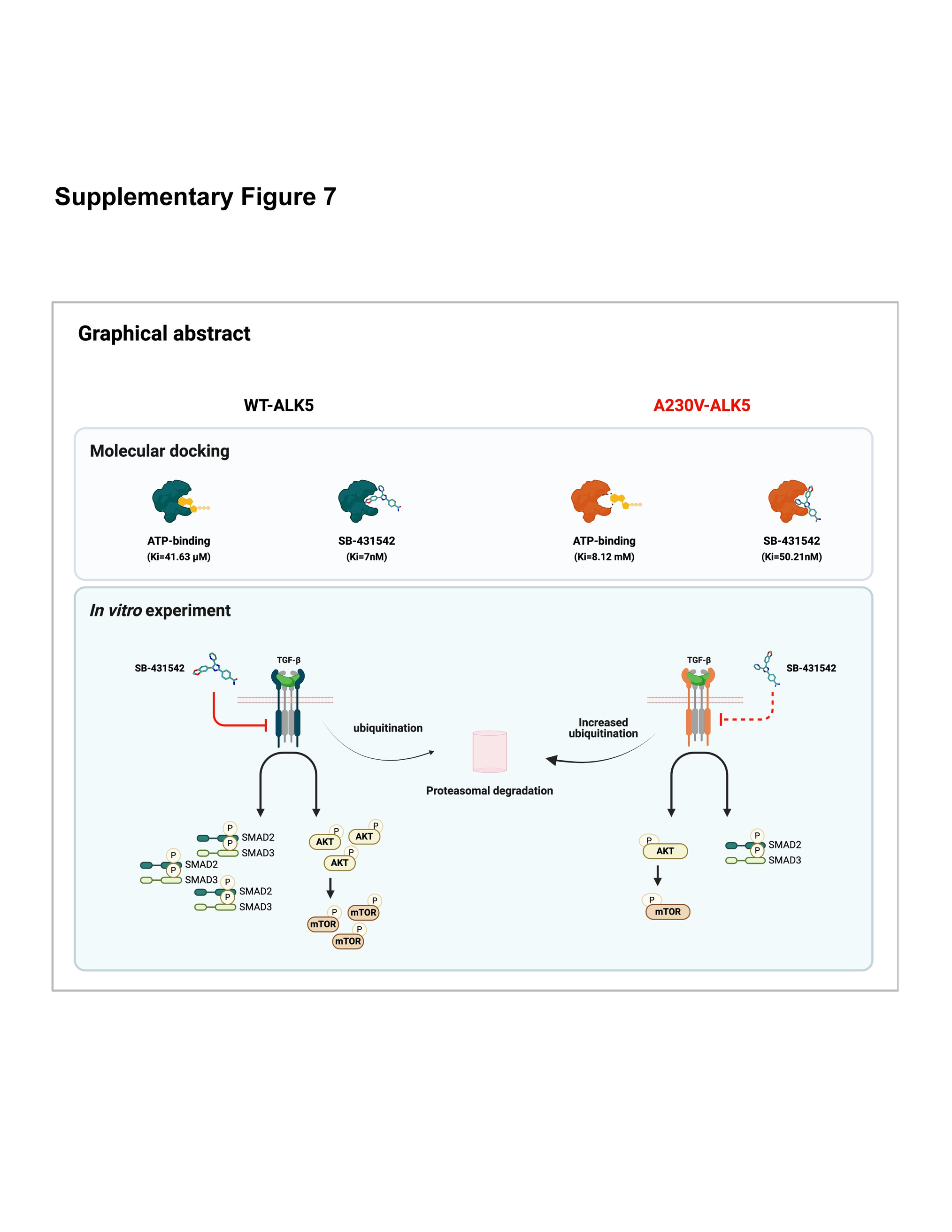

Supplement: S7 Fig — (Top) Molecular docking with ALK5 protein with their ligand, ATP, and SB-431542. (Bottom) In vitro experiment results show the A230V-ALK5 mutant cells show a decrease in both canonical SMAD2/3 and non-canonical signaling and inhibitor sensitivity but show an increase in proteasomal degradation of the receptor protein. (TIF) [file pone.0312806.s015.tif]
