# Supplementary material for: The endometrial cancer A230V-ALK5 (TGFBR1) mutant attenuates TGF-β signaling and exhibits reduced in vitro sensitivity to ALK5 inhibitors
Source: PLoS One. 2024 Nov 22;19(11):e0312806. doi: 10.1371/journal.pone.0312806 (PMC11584080; doi:10.1371/journal.pone.0312806)
Supplement: S1 Table — (DOCX) [file pone.0312806.s016.docx]

**Supplementary Table 1. Interaction, van der Waals, electrostatic and hydrogen bond energies of protein-ligand complex calculated by Autodock Vina**

| **Binding Energy** | | **WT-ALK5** | **A230V-ALK5** |
| --- | --- | --- | --- |
| **ALK5 + SB-431542** | |  |  |
|  | Total Intermolecular Interaction Energy[1] | - 11.0765 kcal/mol | - 9.9015 kcal/mol |
|  | Total Intermolecular vdw + Hbond + desolve Energy[2] | - 10.7626 kcal/mol | - 9.7606 kcal/mol |
|  | Total Intermolecular Electrostatic Energy[3] | - 0.3139 kcal/mol | - 0.1409 kcal/mol |
|  | Total Intermolecular Intramolecular Energy[4] | - 12.3188 kcal/mol | - 11.1511 kcal/mol |
|  | Estimated Free Energy of Binding | - 11.13 kcal/mol | - 9.96 kcal/mol |
|  | Estimated Inhibition Constant, Ki | 7 nM | 50.21 nM |
| **ALK5 + ATP** | |  |  |
|  | Total Intermolecular Interaction Energy[1] | - 7.4009 kcal/mol | - 4.4920 kcal/mol |
|  | Total Intermolecular vdw + Hbond + desolve Energy[2] | - 7.9061 kcal/mol | - 4.0049 kcal/mol |
|  | Total Intermolecular Electrostatic Energy[3] | 0.5052 kcal/mol | - 0.4870 kcal/mol |
|  | Total Intermolecular Intramolecular Energy[4] | - 10.4507 kcal/mol | - 7.3262 kcal/mol |
|  | Estimated Free Energy of Binding | - 5.98 kcal/mol | - 2.85 kcal/mol |
|  | Estimated Inhibition Constant, Ki | 41.63 μM | 8.12 mM |

*Abbreviations: Hbond, hydrogen bond; vdw, van der Waals

*Estimated Free Energy of Binding= [1]+[2]+[3]-[4]
