# Supplementary material for: The endometrial cancer A230V-ALK5 (TGFBR1) mutant attenuates TGF-β signaling and exhibits reduced in vitro sensitivity to ALK5 inhibitors
Source: PLoS One. 2024 Nov 22;19(11):e0312806. doi: 10.1371/journal.pone.0312806 (PMC11584080; doi:10.1371/journal.pone.0312806)
Supplement: S2 Table — (DOCX) [file pone.0312806.s017.docx]

**Supplementary Table 2. MSI status and mutation status of nine EC-associated genes in the**

**ALK5-A230V-mutated endometrial tumor and the HEC-265 EC cell line**

|  | **ALK5-mutated endometrial tumor** | **HEC-265 EC cell line** |
| --- | --- | --- |
| MSI | MSI | MSI |
| *POLE* (exonuclease domain) | Non-mutated | Non-mutated |
| *POLE* (other regions) | Mutated | Mutated |
| *POLD1* (exonuclease domain) | Non-mutated | Non-mutated |
| *POLD1* (other regions) | Mutated | Mutated |
| TP53 | Non-mutated | Non-mutated |
| RPL22 | Mutated | Mutated |
| PTEN | Mutated | Mutated |
| KRAS | Non-mutated | Non-mutated |
| ARID1A | Mutated | Mutated |
| CTCF | Mutated | Mutated |
| PPP2R1A | Mutated | Mutated |
| TGFBR2 | Non-mutated | Non-mutated |

^a^MSI status and mutation data were retrieved from publicly available datasets (Berger et al., 2018; Ghandi et al., 2019) using the cBIO Portal for Cancer Genomics (Cerami et al., 2012; Gao et al., 2013)
